# TBscreen: A passive cough classifier for tuberculosis screening with a controlled dataset

**DOI:** 10.1126/sciadv.adi0282

**Published:** 2024-01-03

**Authors:** Manuja Sharma, Videlis Nduba, Lilian N. Njagi, Wilfred Murithi, Zipporah Mwongera, Thomas R. Hawn, Shwetak N. Patel, David J. Horne

**Affiliations:** ^1^ Department of Electrical and Computer Engineering, University of Washington, 185 E Stevens Way NE, Seattle, WA 98195, USA.; ^2^ Centre for Respiratory Diseases Research, Kenya Medical Research Institute, Mbagathi Rd, Nairobi 610101, Kenya.; ^3^ Department of Medicine, University of Washington, 1959 NE Pacific Street, Seattle, WA 98195, USA.; ^4^ Paul G. Allen School of Computer Science and Engineering, University of Washington, 185 E Stevens Way NE, Seattle, WA 98195, USA.

## Abstract

Recent respiratory disease screening studies suggest promising performance of cough classifiers, but potential biases in model training and dataset quality preclude robust conclusions. To examine tuberculosis (TB) cough diagnostic features, we enrolled subjects with pulmonary TB (*N* = 149) and controls with other respiratory illnesses (*N* = 46) in Nairobi. We collected a dataset with 33,000 passive coughs and 1600 forced coughs in a controlled setting with similar demographics. We trained a ResNet18-based cough classifier using images of passive cough scalogram as input and obtained a fivefold cross-validation sensitivity of 0.70 (±0.11 SD). The smartphone-based model had better performance in subjects with higher bacterial load {receiver operating characteristic–area under the curve (ROC-AUC): 0.87 [95% confidence interval (CI): 0.87 to 0.88], *P* < 0.001} or lung cavities [ROC-AUC: 0.89 (95% CI: 0.88 to 0.89), *P* < 0.001]. Overall, our data suggest that passive cough features distinguish TB from non-TB subjects and are associated with bacterial burden and disease severity.

## Teaser

Passive tuberculosis cough is associated with bacterial burden and differs from coughs in other respiratory conditions.

## INTRODUCTION

Tuberculosis (TB), caused by inhalation of *Mycobacterium tuberculosis* (Mtb), is the second leading infectious disease–related cause of death after coronavirus disease 2019 (COVID-19) (*1*). After years of decline, the estimated incidence of TB and TB-related deaths increased in 2021, numbering 10.6 million and 1.6 million people, respectively. The current gold standards for TB diagnosis include sputum culture or GeneXpert molecular tests (*2*–*5*). The availability of these tests is limited in low resource settings, particularly at peripheral health centers (*6*–*8*). Given its ease of implementation, the World Health Organization (WHO) recommends symptom screening (assessing for the presence of fever, cough, night sweats, or weight loss) to identify people suspected of having TB. Unfortunately, the accuracy of symptom screening is suboptimal in both people with and without HIV (*9*, *10*). The WHO target product profile (TPP) for TB triage tests includes a test that is non–sputum-based, rapid, low-cost, easy to use with minimal infrastructure requirements, and accurate (>90% sensitive, >70% specific) (*11*, *12*). Currently available TB screening tests do not meet these criteria (*13*–*22*).

The mechanism of cough production varies according to mucus properties, respiratory muscle strength, mechanosensitivity, chemosensitivity of airways, and other factors resulting in diverse cough sounds (*23*–*26*). Although prolonged cough is one of the cardinal symptoms of pulmonary TB and one of the main mechanisms of spreading TB (*27*–*30*), current screening methods only include patient self-report to identify cough. The poor sensitivity (51%) of using cough to screen for TB may be due, in part, to lack of patient awareness (*9*). Objective tools to estimate the number of coughs and its characteristics may be useful to improve the sensitivity of cough for TB screening (*27*, *31*–*36*).

Cough audio frequency and its time domain features (*37*) have been studied as a biomarker for several pulmonary diseases, including asthma (*38*), pneumonia (*39*), and TB (*40*). COVID-19 cough diagnostic studies included machine learning approaches with classical modeling tools like logistic regression and support vector machine (*41*) as well as modern deep learning tools like convolutional neural networks (*42*) and transformers (*43*), with sensitivity ranging between 0.65 and 0.98 and specificity between 0.69 and 0.97 (*41*–*44*) in classifying COVID-19 versus non–COVID-19 coughs. For classification of TB, previous work suggests that forced (voluntary) cough features can distinguish TB from other respiratory illnesses with moderate sensitivity and specificity (*45*, *46*).

Despite encouraging performance metrics for several cough-based disease classifiers, cough feature–based screening models have not been reproducible or translatable (*47*, *48*). Several potential reasons for these challenges and knowledge gaps have been highlighted (*31*, *42*), including differences in environmental noise between cases and controls as contributing to the classifier (*41*), small numbers of coughs and participants (*45*), dataset imbalances in participant characteristics between cases and controls (*49*), and inconsistencies in training methods (*43*), which can lead to overestimated metrics (*48*). In addition, it is unclear if forced coughs, which have been used to train the cough classifiers (*50*–*52*), are a correct representation of natural (passive) coughs. Together, these issues highlight that it is important not only to focus on the performance metric but also to critically analyze the features/biases that affect the model training and evaluation.

To investigate cough characteristics as an accurate classifier of TB versus non-TB–related cough, we enrolled adults with cough due to pulmonary TB and non-TB–related etiologies in Nairobi, Kenya. We also investigated whether forced (voluntary) coughs could discriminate between TB disease states. We constructed a study design with minimal background noise and environmental variability between the control (non-TB) and disease groups (TB) to ensure that the model trains on differences in cough features rather than ambient noise. In addition, we used three recording devices with a demographically balanced cohort in a single setting to record a large number of passive (nonforced) coughs. We collected this dataset and trained a binary cough classifier, TBscreen, that provides an opportunity to examine whether there are discriminatory features in the frequency content of TB coughs in comparison to other respiratory diseases and among various TB presentation.

## RESULTS

**Fig. 1.**
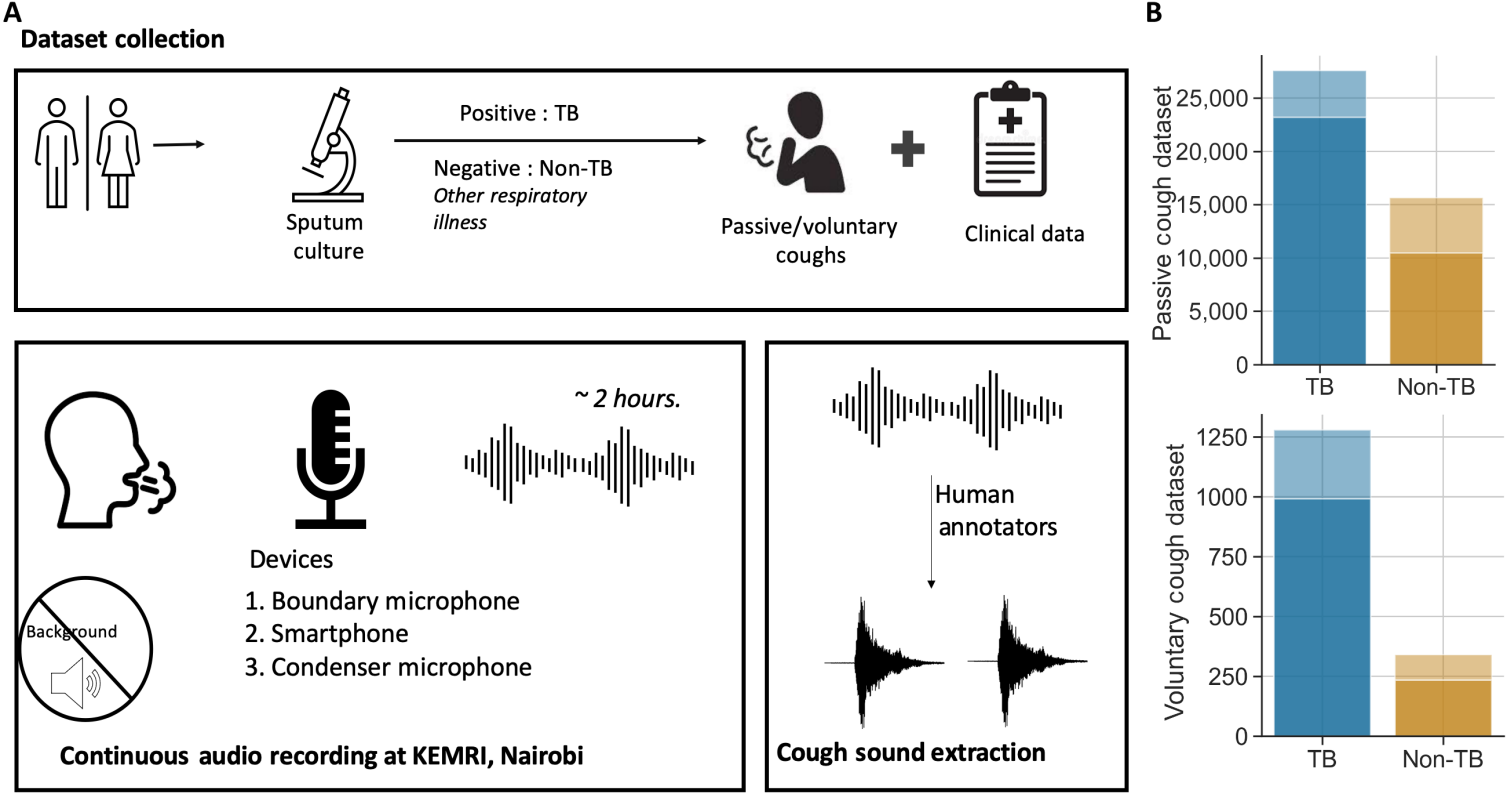
Dataset summary. (**A**) Study protocol for the audio data collection at Kenya Medical Research Institute (KEMRI), Nairobi and subsequent cough annotation at the University of Washington, Seattle. Subjects with tuberculosis (TB) and a control group of subjects having pulmonary symptoms other than TB (non-TB) had natural cough sounds (passive coughs) recorded using three recording devices in a quiet room for 2 hours. A subset of the subjects provided forced coughs (voluntary coughs) at the beginning of each audio recording. These recordings were annotated using Audacity software, and cough sounds with minimum background noise or distortion were selected. (**B**) The bar graphs represent the total passive and voluntary coughs (including all recording devices) in the Nairobi cough dataset. The lighter shade in the bar graphs indicates cough discarded because of environmental noise or audio distortion, and the darker shade represents the selected coughs per group. The adjacent boxplot represents distribution of total selected cough counts per subject including all three recording devices.

To examine whether cough counts or features are TB-specific, we enrolled participants with cough seeking health care who were diagnosed with TB (*N* = 103) and without TB (*N* = 46; Table 1) in Nairobi, Kenya. Participants with TB were recruited from National Treatment Program clinics and were GeneXpert (MTB/RIF or MTB/RIF Ultra) positive, subsequently confirmed by acid-fast bacilli (AFB) culture. All study assessments were performed before the initiation of anti-TB therapy. Participants with non-TB–related cough were recruited from the same National Treatment Program clinics or other clinics and were all GeneXpert negative, had chest x-rays not compatible with TB, and were determined to have cough due to conditions other than TB (e.g., bacterial pneumonia, viral upper respiratory infection, and asthma) by study clinicians. Both groups had similar demographic and clinical characteristics, with many subjects self-reporting cough for longer than 2 weeks, fever, and night sweats (Table 1). The median age of persons with TB was 36 years [interquartile range (IQR), 27 to 42] and of those with non-TB–related cough was 40 years (IQR, 33 to 45). We extracted 43,200 passive (natural) coughs from these subjects (*N* = 149) by annotating continuous 2-hour audio recordings from three devices used simultaneously (smartphone, low-cost boundary microphone, and high-cost condenser microphone) in a dedicated and relatively quiet room (Fig. 1A).

**Table 1.** Demographic and clinical information of cohorts.

			Full cohort (T2)		Training and evaluation cohort (T1)		Forced cough cohort (T3)	
	Category	Subcategory	TB	Non-TB	TB	Non-TB	TB	Non-TB
Total subjects	–	–	103	46	45	45	29	8
	Recruitment	Pulmonary TB (cohort A)	103 (100%)	–	45 (100%)	–	29 (100%)	–
		Non-TB (failed cohort A)	–	36 (78%)	–	35 (77%)	–	2 (25%)
		Non-TB (co hort C)	–	10 (22%)		10 (22%)		6 (75%)
Demographic	Gender	Male	75 (73%)	27 (57%)	27 (60%)	27 (60%)	21 (73%)	3 (38%)
	Age group	(18–40)	69 (67%)	23 (50%)	33 (73%)	23 (51%)	21 (72%)	5 (63%)
		(40–60)	31 (30%)	18 (40%)	11 (24%)	18 (40%)	7 (24%)	2 (25%)
		Median age	36 years	40 years	33 years	40 years	39 years	32 years
Clinical history	HIV history	Yes	12 (12%)	16 (35%)	5 (11%)	16 (36%)	0	3 (36%)
	Smoking history	Yes	41 (40%)	5 (10%)	12 (27%)	5 (11%)	12 (41%)	1 (13%)
	Coughing status (any duration)	Coughing	95 (92%)	41 (89%)	41 (91%)	41 (91%)	26 (90%)	6 (75%)
	Coughing duration (>2 weeks)	Yes	88 (85%)	35 (76%)	40 (89%)	35 (78%)	25 (86%)	6 (75%)
	Hemoptysis	Yes	29 (28%)	10 (22%)	12 (27%)	10 (22%)	7 (24%)	6 (75%)
	Fever	Yes	76 (74%)	23 (50%)	39 (87%)	23 (51%)	18 (62%)	2 (25%)
	Weight loss	Yes	89 (86%)	27 (59%)	38 (84%)	27 (60%)	25 (86%)	4 (50%)
	Night sweats	Yes	77 (75%)	17 (37%)	35 (78%)	17 (38%)	18 (62%)	3 (38%)
	Comorbidity	Diabetes	3 (3%)	–	3 (6%)	–	0	–
		Asthma	1 (1%)	–	1 (2%)	–	0	–
		Prior TB history	15 (15%)	11 (24%)	5 (11%)	11 (24%)	3 (10%)	2 (25%)
	Chest x-ray findings	Cavitary disease	74 (72%)	0	32 (71%)	0	7 (24%)	0
		Lung consoli dation	55 (53%)	2 (4%)	22 (49%)	2 (4%)	6 (21%)	1 (13%)
		Abnormal lung quadrants	98 (95%)	4 (8%)	39 (87%)	4 (8%)	11 (34%)	1 (13%)
		Normal	0	27 (57%)	0	27 (60%)	0	4 (50%)
TB presentations	GeneXpert	Negative	0		0		0	
		Trace	6 (6%)		4 (9%)		4 (14%)	
		Very low	7 (7%)		4 (9%)		1 (3%)	
		Low	19 (18%)		8 (18%)		8 (38%)	
		Medium	40 (39%)		14 (31%)		8 (28%)	
		High	30 (29%)		15 (33%)		8 (28%)	
	Sputum smear	Negative	3 (3%)		1 (2%)		0	
		Scanty	4 (4%)		2 (4%)		1 (3%)	
		1+	24 (23%)		10 (22%)		4 (14%)	
		2+	26 (25%)		7 (16%)		8 (28%)	
		3+	39 (38%)		21 (47%)		11 (38%)	

To examine the relationship of cough features between participants with TB and non-TB–related cough, we selected coughs with minimum background noise and audio distortion for analysis, bringing the total clean passive cough count to 33,641. In addition, we collected forced coughs from a subset of participants (42 TB and 8 non-TB), giving us a total of 1619 coughs from all three recording devices (Fig. 1B). Among these, 1225 coughs with minimum background noise or audio clippings were selected for cough feature analysis. The Nairobi dataset provides a unique opportunity to evaluate the feasibility of a cough-based screening tool by comparing disease (TB) and control (non-TB) cough features, recorded with low demographic variability and minimum ambient noise interference.

### Cough counts and TB presentation

**Fig. 2.**
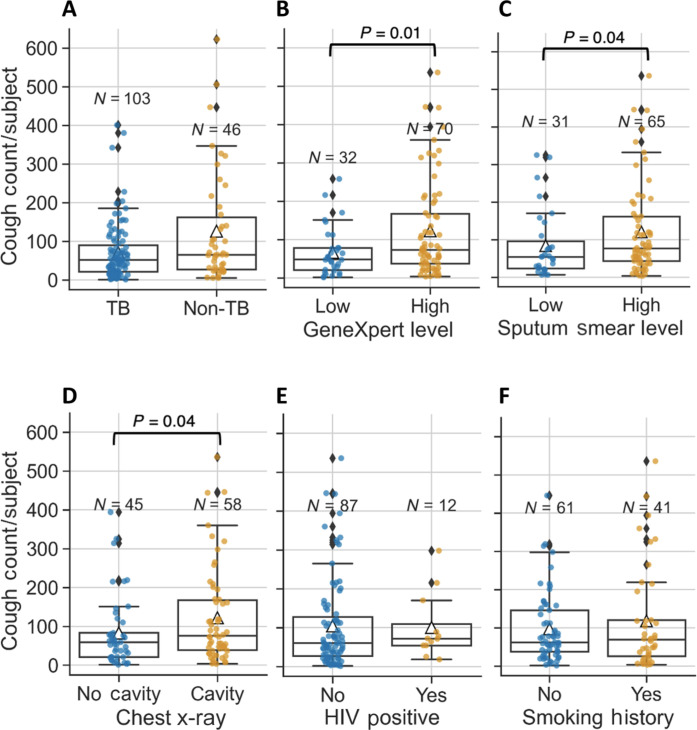
Summary of cough counts in a 2-hour interval. Box plots in (**A**) represent cough distribution of TB versus non-TB subjects. Mean number of coughs in each box plot is depicted by a triangle. Similarly, cough distribution for various subcategories of TB subjects is summarized with (**B**) low and high polymerase chain reaction (PCR) test result using GeneXpert, (**C**) low or high sputum smear score, (**D**) with or without cavity on chest x-ray findings, (**E**) with or without HIV infections, and (**F**) with or without a history of smoking. In each graph, the total number of subjects in the category is shown by *N*. Data for subjects with missing subcategory information are not shown. *P < 0.05 with univariate testing by Mann-Whitney *U* statistical test using “greater” alternative.

We first examined whether passive cough counts were associated with TB compared to those without TB-related cough. Median cough counts in participants with TB were similar to those without TB (64 versus 65, coughs, *P* = 0.64; Fig. 2A). We also evaluated whether cough count was associated with different TB presentations. Increased cough counts were associated with an increase in sputum Mtb bacterial load measured by GeneXpert [low (48 coughs) and high (73 coughs), *P* = 0.01; Fig. 2B] and sputum AFB smear [low (54 coughs) or high (76 coughs) sputum smear, *P* = 0.04; Fig. 2C]. Participants with lung cavities on chest radiographs (76 coughs) had higher median cough counts compared to those without lung cavities (59 coughs; *P* = 0.04; Fig. 2D). The median number of coughs was similar in TB subjects with and without HIV infection (72 versus 60 coughs, *P* = 0.50) and with and without history of smoking (69 versus 62 coughs, *P* = 0.93) (Fig. 2, E and F). Using a generalized linear regression model to test associations between clinical variables and cough counts, we found that only semiquantitative grading of GeneXpert remained significant with an increase of 22.8 counts with each additional GeneXpert grade (*P* = 0.02, additional data in table S1). The addition of other variables to this model did not significantly improve its performance. Overall, our data suggest that participants have similar cough counts regardless of cough etiology. Among participants with pulmonary TB, higher Xpert semiquantitative grade was associated with greater cough counts.

### Binary cough classifier performance across different test sets, cough types, and devices

**Fig. 3.**
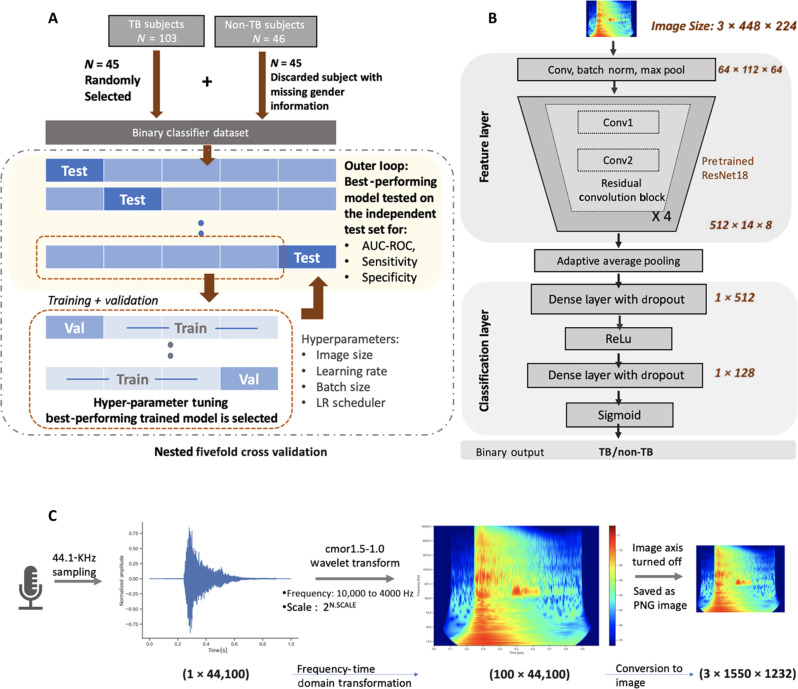
Binary TB/non-TB cough classifier. (**A**) A balanced subset of the Nairobi dataset is used for fivefold nested cross-validation. (**B**) Resnet18 model architecture and feature dimension size used to train/test the cough classifier. (**C**) Preprocessing of cough sound into an RGB scalogram image, which is used as input features to the Resnet18 model.

**Fig. 4.**
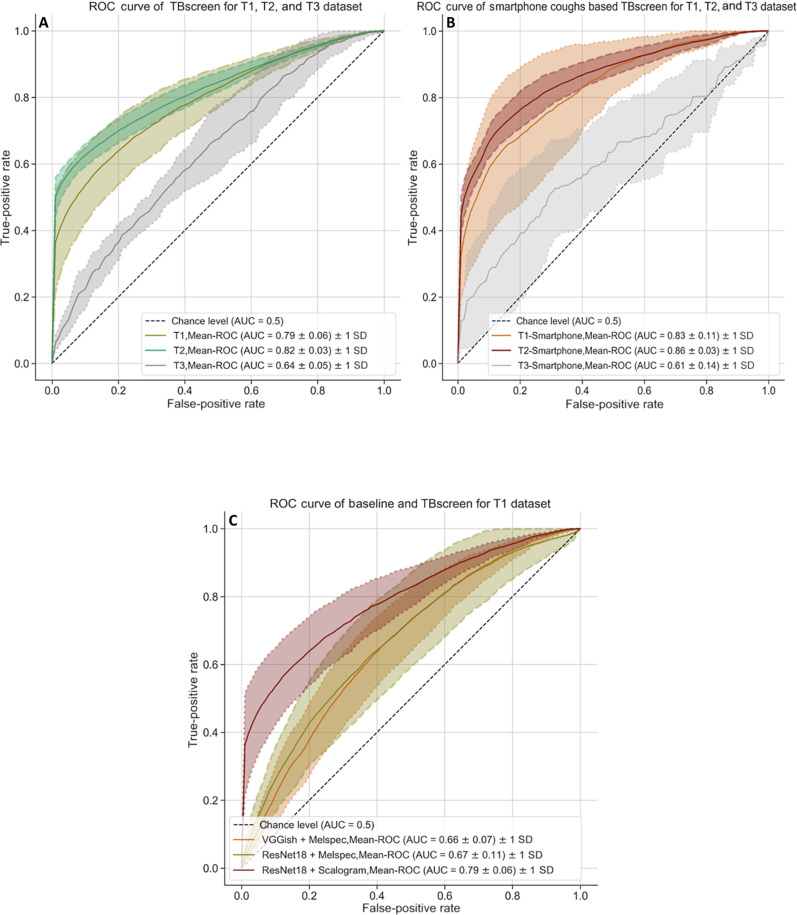
Passive binary cough ROC plot. (**A**) ROC curve with SD across five folds for model trained using coughs from all devices and evaluated on T1: subject balanced passive cough dataset (equal gender distribution and number of subjects) and used for fivefold training and testing of the classifier; T2: expanded T1 consisting of all non-TB subjects and TB cough data not included for training the fivefold classifier; T3: a voluntary cough dataset consisting of coughs from TB and non-TB subjects. ROC curve with SD for the second model trained on and validated on coughs from smartphone is also represented (**B**) ROC curve with SD across five folds for model trained using coughs from smartphone and evaluated on T1, T2, and T3. (**C**) Comparison of ROC curve of the binary cough classifier trained using scalogram images of cough and baseline cough models trained on mel-spectrogram features. Figure S2 represents aggregated results from multiple folds using bootstrapping.

We next examined whether spectral features of cough audio distinguished TB from non-TB–related coughs. We developed TBscreen, a ResNet18 (*53*)–based TB versus non-TB classifier using RGB images of scalogram [time-frequency feature map generated using complex Morlet wavelet transform (*54*)] of passive cough sounds from all three recording devices. The model was trained and tested using fivefold cross-validation (Fig. 3, A to C) (*55*, *56*) on dataset T1 (*N* = 90: TB = 45, non-TB = 45; Table 1 and fig. S1A). Ninety subjects were randomly divided into five groups called folds, with each fold having a balanced number of unique subjects (subject-independent folds) and identical gender distribution between the two classes (TB and non-TB; fig. S1A). For fivefold cross-validation, the binary classifier was first trained and validated on four of the five folds and then evaluated on the independent reserved fold, with the entire process repeated four times (Fig. 3A). On evaluation, the ResNet18 classifier, TBscreen (Fig. 3B), generated an average receiver operating characteristic–area under the curve (ROC-AUC) of 0.79 and an SD of 0.06 (sensitivity: 0.70 ± 0.11, specificity: 0.71 ± 0.10) across five folds on the subject balanced dataset T1 (Table 2, ROC curve for T1 with SD across different folds in Fig. 4A). The five test folds of T1 dataset were expanded to form T2 (*N* = 149: TB = 103, non-TB = 46; fig. S1B) by including all non-TB (*n* = 1) and TB (*n* = 58) data in the Nairobi dataset that was not used for balanced classifier training/evaluation. The model results on dataset T2 across five folds had an ROC-AUC score of 0.82 ± 0.03 (sensitivity: 0.74 ± 0.02, specificity: 0.72 ± 0.10; Table 2; ROC curve in Fig. 4A). Additional aggregated statistics from five folds are summarized in fig. S2 and table S3. Cross-validation results indicate that a scalogram-based model can differentiate between TB and non-TB passive coughs across all folds.

**Table 2.** Performance across datasets. Average ROC-AUC score, sensitivity, and specificity with SD across five folds using different training data (coughs from all devices versus coughs from smartphone) and test sets (T1, T2, and T3). Two variations of the classifier are tested on three different test sets: T1: subject balanced passive cough dataset (for gender and number of subjects) and used for fivefold training and testing of the classifier; T2: expanded T1 consisting of all non-TB subjects and TB cough data not included for training the fivefold classifier; T3: a voluntary cough dataset consisting of coughs from TB and non-TB subjects. Table S2 represents aggregated result from multiple folds using bootstrapping.

	Model training parameters	Test set	ROC-AUC score (average of 5 folds ± SD across folds)	Sensitivity (average of 5 folds ± SD across folds, threshold = 0.5)	Specificity (average of 5 folds ± SD across folds, threshold = 0.5)	Sensitivity at 70% specificity (average of 5 folds ± SD across folds)	Combined ROC- AUC score of 5 folds (average after combining results from all five folds (DeLong’s CI)
TBscreen	Device: All Scalo gram: 10 Hz to 4 kHz Sampling rate: 44.1 kHz	T1: Subject balanced CV	0.79 ± 0.06	0.70 ± 0.11	0.71 ± 0.10	0.72 ± 0.10	0.80 (0.79–0.80)
		T2: Expanded T1, unbalanced set	0.82 ± 0.03	0.74 ± 0.02	0.72 ± 0.10	0.76 ± 0.04	0.80 (0.79–0.80)
		T3: Voluntary cough, unbal anced set	0.64 ± 0.05	0.34 ± 0.13	0.81 ± 0.12	0.47 ± 0.06	0.64 (0.62–0.66)
TBscreen trained/ evaluated on coughs from smartphone	Device: Smart phone Scalogram: 10 Hz to 4 kHz Sampling rate: 44.1 kHz	T1 subset: Sub ject balanced CV	0.83 ± 0.11	0.76 ± 0.12	0.74 ± 0.10	0.76 ± 0.20	0.85 (0.84–0.85)
		T2 subset: Expanded T1, unbalanced set	0.86 ± 0.03	0.80 ± 0.03	0.74 ± 0.10	0.83 ± 0.05	0.86 (0.85–0.87)
		T3 subset: Voluntary cough, unbalanced set	0.61 ± 0.14	0.16 ± 0.11	0.95 ± 0.05	0.51 ± 0.18	0.66 (0.62–0.70)

We next performed several secondary analyses. To understand whether the model trained on passive coughs can analyze forced coughs, we evaluated the classifier’s performance on a test set consisting of only forced coughs (dataset T3, TB = 29, non-TB = 8; fig. S1C). The classifier’s sensitivity dropped to 0.34 ± 0.13, while specificity increased to 0.81 ± 0.12 with an ROC-AUC score of 0.64 ± 0.05. This indicated that the forced cough model performed poorly and classified the majority of forced cough as non-TB (Table 2 and table S2).

We also examined whether there were audio recording device–related performance differences. We used a subset of the cough dataset by including coughs from only one recording device to train and evaluate the model performance on the T1 and T2 subsets. The smartphone-based model performed best with 0.83 ± 0.11 ROC-AUC for T1 subset and 0.86 ± 0.03 for T2 subset (Tables 2 and 3 and tables S3 and S4). Because our dataset contains multiple coughs from the same participant, along with evaluating performance per cough, we evaluated the model for accuracy per participant. The model on an average correctly classified TB coughs per TB participant with an accuracy of 0.68 [95% confidence interval (CI): 0.61 to 0.75, *N* = 103; table S8] and 0.78 (95% CI: 0.70 to 0.86, *n* = 45) for non-TB coughs per non-TB subject. Together, these data demonstrate that TBscreen can discriminate between TB and non-TB cough scalograms, but a model trained on passive cough is not translatable to predict using forced cough scalograms. The model based on smartphone coughs had the best performance.

**Table 3.** Various cough features and model performance. Average ROC-AUC score, sensitivity, and specificity with SD across five folds using different training inputs and performance evaluated on T1 dataset. Table S5 represents aggregated result from multiple folds using bootstrapping.

	Model training parameters	Test set	ROC-AUC score (average of 5 folds ± SD across folds)	Sensitivity (aver age of 5 folds ± SD across folds, threshold = 0.5)	Specificity (average of 5 folds ± SD across folds, threshold = 0.5)	Sensitivity at 70% specificity (average of 5 folds ± SD across folds)	Combined ROC- AUC score of 5 folds (average after combin ing results from all 5 folds (DeLong’s CI)
Frequency range of scalogram Device: All Sampling rate: 44.1 kHz	10 Hz to 4 kHz	T1: Subject balanced CV	0.79 ± 0.06	0.70 ± 0.11	0.71 ± 0.10	0.72 ± 0.10	0.80 (0.79–0.80)
	4–8 kHz	T1: Subject balanced CV	0.75 ± 0.09	0.64 ± 0.19	0.68 ± 0.08	0.64 ± 0.14	0.75 (0.75–0.76)
	10–16 kHz	T1: Subject balanced CV	0.79 ± 0.07	0.72 ± 0.07	0.69 ± 0.10	0.71 ± 0.12	0.80 (0.79–0.80)
Sampling rate Device: All	8 kHz Scalogram: 10 Hz to 4 kHz	T1: Subject balanced CV	0.65 ± 0.09	0.64 ± 0.18	0.57 ± 0.1	0.51 ± 0.14	0.63 (0.62–0.63)
	44.1 kHz scalo gram: 10 Hz to 4 kHz	T1: Subject balanced CV	0.79 ± 0.06	0.70 ± 0.11	0.71 ± 0.10	0.72 ± 0.10	0.80 (0.79–0.80)
Recording device Scalogram: 10 Hz to 4 kHz Sampling rate: 44.1 kHz	Smartphone	T1 subset: Subject balanced CV (smartphone coughs)	0.83 ± 0.11	0.76 ± 0.12	0.74 ± 0.10	0.76 ± 0.20	0.85 (0.84–0.85)
		T1: Subject balanced CV	0.79 ± 0.03	0.76 ± 0.05	0.62 ± 0.04	0.72 ± 0.06	0.80 (0.79–0.80)
	Boundary micro phone	T1 subset: Subject balanced CV (boundary microphone coughs)	0.77 ± 0.10	0.69 ± 0.09	0.67 ± 0.20	0.69 ± 0.13	0.78 (0.77–0.78)
		T1: Subject balanced CV	0.77 ± 0.09	0.72 ± 0.12	0.66 ± 0.14	0.68 ± 0.14	0.79 (0.79–0.80)
	Condenser microphone	T1 subset: Subject balanced CV (condenser microphone coughs)	0.73 ± 0.14	0.65 ± 0.19	0.65 ± 0.13	0.62 ± 0.22	0.73 (0.72–0.74)
		T1: Subject balanced CV	0.69 ± 0.17	0.67 ± 0.18	0.53 ± 0.18	0.56 ± 0.25	0.69 (0.68–0.69)
Alternative frequency representation Mel-spectrogram Device: All Baseline model	Model: VGGish Sampling rate: 16 kHz	T1: Subject balanced CV	0.66 ± 0.07	0.62 ± 0.19	0.61 ± 0.09	0.53 ± 0.12	0.63 (0.63–0.64)
	Model: ResNet18 Sampling rate: 44.1 kHz	T1: Subject balanced CV	0.67 ± 0.11	0.66 ± 0.16	0.58 ± 0.10	0.55 ± 0.16	0.65 (0.65–0.66)

### Association of cough features with TB clinical presentation

We extended the binary classification model (TB versus non-TB) to a multiclass classifier to examine whether cough features can differentiate between non-TB and various levels of clinical presentations of TB. The model included three distinct classes: non-TB (class 0), low TB burden presentation (class 1), and high TB burden presentation (class 2). The level of TB burden was based either on Mtb bacillary load estimated using GeneXpert or sputum smear result (low versus high bacterial load) or on the presence (high)/absence (low) of lung cavities. Therefore, three different models based on GeneXpert, sputum smear, and chest x-ray were trained and evaluated using fivefold cross-validation. Each fold had coughs from unique participants (subject independent), balanced number of participants, and equal gender distribution across three classes (fig. S2). The accuracy score for a model based on GeneXpert was 0.40 ± 0.09 (*n* = 90; per class subjects = 30), sputum smear 0.45 ± 0.05 (*n* = 105; per class subjects = 35), and chest x-ray 0.44 ± 0.03 (*n* = 117; per class subjects = 39). All three models had the highest sensitivity for class 0 (GeneXpert: 0.58 ± 0.23, sputum smear: 0.58 ± 0.05, chest x-ray: 0.62 ± 0.07) and lowest sensitivity for class 1 (GeneXpert: 0.21 ± 0.08, sputum smear: 0.33 ± 0.15, chest x-ray: 0.23 ± 0.08). In summary, the accuracy and sensitivity of all three multiclass models were suboptimal and had lowest performance in distinguishing between class 1 and class 2 (further details are provided in table S9 and fig. S3). Of the three models, chest x-ray had the best sensitivity for all three classes.

### Impact of cough audio frequency, sampling rate, devices, and audio frequency representations on binary model performance

We examined various characteristics of recorded audio, recording device, and feature representation that affects the model to select the criteria for best performance. First, we analyzed the impact of various cough audio characteristics on model performance by training and testing cough classifiers (dataset T1) using scalogram generated from different audio frequency ranges (10 Hz to 4 kHz, 4 to 8 kHz, and 10 Hz to 16 kHz) and sampling rates. The model performed best in the frequency range of 10 Hz to 4 kHz (sensitivity: 0.70 ± 0.11, specificity: 0.71 ± 0.10) and had lower sensitivity and specificity in frequency ranges above 4 kHz (0.64 ± 0.19, 0.68 ± 0.08, *P* < 0.001; Table 3 and tables S5 and S10). Next, we analyzed audio features using different sampling rates (rate at which audio data are sampled by the device: 8 and 44.1 kHz) to verify its impact on the model performance. We observe that the model performance degrades with a lower sampling rate (*P* < 0.001; Table 3 and tables S5 and S10). The binary cough model performs best in the lower frequency range of human ear (10 Hz to 4 kHz) and has better performance for higher audio sampling rate (44.1 kHz).

Second, we assessed the influence of recording devices by training and evaluating device-specific cough classification models. The cough model was trained and tested on each fold in T1 using data only from a specific recording device (T1 subset), scalogram features in frequency range of 10 Hz to 4 kHz, and audio sampling rate of 44.1 kHz. Overall, the model trained with smartphone data performed best with an average AUC-ROC score of 0.83 ± 0.1, sensitivity of 0.76 ± 0.12, and specificity of 0.74 ± 0.1, followed by boundary microphone (sensitivity: 0.69 ± 0.09, specificity: 0.67 ± 0.20, *P* < 0.001), and then condenser microphone (sensitivity: 0.65 ± 0.19, specificity: 0.65 ± 0.13, *P* < 0.001) (Fig. 4B, Table 3, and table S10). On evaluating device-specific models on complete T1 test set (including coughs from all the recording device), we found a drop in specificity across all device models. Results indicate that the smartphone-based model performs best in comparison to the other two recording devices, and a model trained on any one device has lower performance while evaluating cough data from multiple recording devices.

Last, we compared common audio classification model architectures, VGGish *(**57**)* and ResNet18 models trained on mel-spectrogram (alternative audio frequency representations) as a baseline against the scalogram model. The mel-spectrogram feature set was able to classify TB/non-TB coughs (AUC-ROC score: 0.61 ± 0.06/0.62 ± 0.08, VGGish/ResNet18) but had lower sensitivity (0.62 ± 0.19/0.66 ± 0.16, VGGish/ResNet18) and specificity (0.61 ± 0.09/0.58 ± 0.10, VGGish/ResNet18) in comparison to TBscreen (AUC-ROC score: 0.79 + 0.06, *P* < 0.001) built using scalogram features (Table 3, Fig. 4C, fig. S2C, and table S6). Together, these subgroup analyses demonstrated that scalogram cough features generated in a frequency range of 10 Hz to 4 kHz from cough audio recorded using smartphone at a sampling rate of 44.1 kHz had the best performance in differentiating between TB and non-TB coughs.

### Performance bias of smartphone-based binary cough model

We further examined the best-performing model (smartphone cough-based model, frequency range: 10 HZ to 4 kHz, and audio sampling rate: 44.1 kHz) within different demographic/clinical subcategories like gender, age, smoking status, HIV infection, and different presentations of TB subjects (Table 4 and table S11) using T1 subset (only coughs recorded with smartphone). The model demonstrated better performance with male coughs (ROC-AUC: 0.87 ± 0.15) over female coughs (ROC-AUC: 0.78 ± 0.12, *P* < 0.001). The model performed better for older age group (ROC-AUC in 18 to 40: 0.80 ± 0.15, 40 to 60: 0.87 ± 0.11, *P* = 0.01). The model performance was also better for people living with HIV compared to subjects without HIV infection (*P* < 0.001). Subjects with a smoking history had higher ROC-AUC score (0.87 ± 0.17, *P* < 0.001) over subjects with no smoking history (0.80 ± 0.10). The ROC-AUC score of the model in differentiating between TB and non-TB coughs was higher for subjects with TB who had a high GeneXpert semiquantitative grade (0.86 ± 0.12, *P* < 0.001) versus those with TB who had low GeneXpert grades (0.69 ± 0.12). The model performed better in subjects with cavitary TB (0.86 ± 0.12, *P* < 0.001) versus no lung cavity on chest x-ray (0.71 ± 0.05). Overall, the smartphone-based cough model had better performance with male subjects and subjects having a higher GeneXpert semiquantitative grade or a cavitary chest x-ray and was unaffected by age.

**Table 4.** Smartphone classification and performance bias analysis of binary cough model. The classification results are represented for different demographic, clinical, and TB presentations in T1 dataset. Table S6 represents aggregated result from multiple folds using bootstrapping.

	Category	Subcategory	ROC-AUC score (average of 5 folds ± SD across folds)	Sensitivity (average of 5 folds ± SD across folds, threshold = 0.5)	Specificity (average of 5 folds ± SD across folds, threshold = 0.5)	Sensitivity @70% speci ficity (average of 5 folds ± SD across folds)	Combined ROC- AUC score of 5 folds (average after combin ing results from all 5 folds (DeLong’s CI)
Overall model device: Smartphone Scalogram: 10 Hz to 4 kHz Sampling rate: 44.1 kHz	All inclusive	–	0.83 ± 0.11	0.76 ± 0.12	0.74 ± 0.10	0.76 ± 0.20	0.85 (0.84–0.85)
Demographic bias	Gender	Male	0.87 ± 0.15	0.85 ± 0.14	0.72 ± 0.13	0.84 ± 0.25	0.89 (0.89–0.90)
		Female	0.78 ± 0.12	0.56 ± 0.13	0.81 ± 0.14	0.70 ± 0.23	0.76 (0.74–0.78)
	Age group	(18,40)	0.80 ± 0.15	0.74 ± 0.16	0.71 ± 0.14	0.70 ± 0.25	0.85 (0.84–0.86)
		(40,60)	0.87 ± 0.11	0.81 ± 0.12	0.78 ± 0.16	0.86 ± 0.16	0.83 (0.81–0.84)
Clinical bias	HIV history	No HIV history	0.82 ± 0.10	0.74 ± 0.13	0.72 ± 0.13	0.77 ± 0.17	0.84 (0.83–0.85)
		With HIV history	0.92 ± 0.08	0.89 ± 0.07	0.81 ± 0.17	0.90 ± 0.10	0.91 (0.90–0.93)
	Smoking history	No smoking history	0.80 ± 0.10	0.71 ± 0.11	0.73 ± 0.10	0.72 ± 0.18	0.81 (0.80–0.83)
		With smoking history	0.87 ± 0.17	0.83 ± 0.15	0.81 ± 0.18	0.83 ± 0.27	0.90 (0.89–0.91)
TB presentations	GeneXpert	Low bacterial load	0.69 ± 0.12	0.52 ± 0.22	0.74 ± 0.1	0.58 ± 0.24	0.74 (0.73–0.76)
		High bacterial load	0.86 ± 0.12	0.82 ± 0.13	0.74 ± 0.1	0.82 ± 0.21	0.87 (0.87–0.88)
	Sputum smear	Low bacterial load	0.84 ± 0.15	0.76 ± 0.21	0.74 ± 0.1	0.78 ± 0.23	0.85 (0.83–0.87)
		High bacterial load	0.85 ± 0.11	0.79 ± 0.12	0.74 ± 0.1	0.80 ± 0.21	0.86 (0.85–0.87)
	Lung cavity	No cavity	0.71 ± 0.05	0.52 ± 0.1	0.74 ± 0.1	0.56 ± 0.13	0.71 (0.69–0.72)
		With cavity	0.86 ± 0.12	0.83 ± 0.12	0.74 ± 0.1	0.83 ± 0.21	0.89 (0.88–0.89)

## DISCUSSION

We investigated whether cough characteristics discriminate between TB and non-TB–related coughs. Although cough counts did not discriminate between cough related to TB versus other conditions, cough scalogram characteristics were associated with identification of coughs due to pulmonary TB. Our initial ResNet18 classifier model distinguished TB versus non-TB coughs with ROC curve value 0.79 ± 0.06 using scalogram features (variation of signal energy over time and frequency) generated in the frequency range of 10 to 4 kHz and sampling rate of 44.1 kHz using a balanced dataset. We found that the best-performing model was based on recordings from a Pixel smartphone (ROC curve 0.83 ± 0.10). Further improvements in accuracy of the smartphone-based model were noted in detecting participants with a high GeneXpert semiquantitative grade, who are likely most infectious, compared to those with non-TB–related cough (ROC-AUC: 0.86, 95% CI: 0.87 to 0.88). This model achieves a sensitivity of 82% (±21%) at 70% specificity, a level that approaches the WHO TPP for a TB triage test of 90% sensitivity and 70% specificity.

While several recent studies have shown similar or greater accuracy in TB cough classifiers (*45*, *46*, *52*), we believe that several strengths of the present study elevate the validity of our findings and support the potential of cough classifiers as TB triage tests. In a recently published longitudinal study by Huddart *et al.* (*36*) comparing cough counts between TB and non-TB subjects having prolonged coughs, there was a difference in median hourly cough counts of TB versus non-TB subjects. At enrollment, they observed a median of eight coughs per hour for persons with microbiologically confirmed TB compared to five coughs per hour for persons with coughs due to other etiologies. In our study, we found a median of 64 and 65 coughs over a 2-hour recording in persons with TB and persons with non-TB etiologies of cough, respectively. The differences in our results could be due to differences in patient populations, TB severity, differences in the etiologies of non-TB–related coughs, clinic versus nonclinic cough measurements, and the limitations of cough counters in identifying user-specific coughs. This can lead to inflated data for participants living in multiperson households (neighborhoods) with more than one person coughing. Three other studies of cough feature–based TB detection recorded forced (voluntary) coughs. In our study, we found that forced coughs performed poorly when applied to a model trained on passive coughs, highlighting differences in these cough types. Two of the prior studies enrolled healthy volunteers as the control group (*45*, *52*), which would not be reflective of the expected clinical role (persons suspected of having pulmonary TB) for cough screening algorithms. Other important limitations to the prior studies include gender imbalance between classes and the presence of significant ambient noise in the dataset (*46*). Additional strengths of our study include rigorous evaluations to exclude TB in non-TB–related controls, cough recordings obtained before TB treatment initiation, strict recording conditions and standardized lengths of recordings (2 hours), and training/evaluation using a gender-balanced dataset (T1).

We assessed both scalogram and mel-spectrogram cough representation methods to assess optimal frequency timescale cough representation. Scalograms generated using continuous wavelet transform provide better frequency versus time resolution compared to other frequency domain transformations (*58*–*60*). This approach has been used in the analysis of other one-dimensional (1D) data like electroencephalogram (*61*), DNA analysis (*62*), and lung auscultation (*63*). The resolution of scalograms comes at the cost of increased feature size, especially for audio data, limiting its applications. To reduce the size of generated scalograms, we used colored (RGB) images as an encoding for scalograms to train TBscreen (*64*, *65*), making it easier to use medium-sized deep learning models for training. We could then leverage pretrained image classification models reducing the need for a very large training dataset. Traditional cough-based disease classifiers have been developed using mel-spectrogram (*41*, *42*, *45*, *46*) by transforming short-time Fourier transform (STFT) spectrograms with mel-filter banks. These filter banks mimic frequency sensitivity of a human ear as well reduces dimension of an STFT-based spectrogram (*66*). It is difficult for clinicians to screen patients by listening to their cough sounds, and mel-spectrograms (based on response to human ears) are a less accurate approach. Our results confirm that a model using scalogram features performs better than mel-spectrogram features in TB disease classification. In addition, impact of various factors (demographic, recording devices, cough features, and TB presentation) provided rigorous performance analysis of the TB cough model.

Models trained using different recording devices show a varied response, with the smartphone-based model having the best performance. The condenser microphone was overly sensitive, resulting in the capture of more ambient sound and a smaller training dataset due to audio distortion. The boundary microphone is a low-cost microphone that might not have captured the cough sound adequately, although its built-in algorithm to reduce ambient sound could have positively affected the performance. Audio recording devices with dynamic gain are ideal for recording coughs for analysis. Narrowing the frequency of interest would help mitigate issues of designing a universal cough model as it can enable development of tools that are sensitive to audio frequencies only in the range of interest.

Our study has several limitations. We found that the scalogram model performed better with males and those with a higher bacillary load and the presence of cavities. Overall, number of female subjects and coughs from female participants [*n* = 36, coughs = 6559 (31%)] were lower than male subjects in the dataset [*n* = 54, coughs = 14,574 (69%)], which could cause the difference in performance for the two genders. In addition, the improved performance in subjects with a higher bacterial load or chest cavity could be attributed to more disease signal in coughs of subjects with advanced TB. The overall study results are limited in terms of number of subjects and needs evaluation with a larger independent dataset. The cough count analysis was limited by the observation time, and robust monitoring for longer duration may detect differences by cough etiology in cough counts. Increasing the training data can possibly improve the present sensitivity of TBscreen, which is currently lower than the WHO’s TPP (sensitivity: 90%, specificity: 70%) for a TB screening tool (*9*). Our study did not include subjects across the entire spectrum of clinical presentation. For example, some individuals are treated for TB based on high clinician suspicion despite negative diagnostic tests. We did not include these subjects to avoid results based on subjective clinician decisions, which could have led to misclassification of cough etiology. Future studies are needed to address patients with a clinical diagnosis of TB. In addition, TBscreen was trained on a controlled dataset and is not optimized for time efficiency or robustness to any ambient noise essential for real-world deployment.

The Nairobi cough dataset provides a unique access to passive coughs of both disease (TB) and control group (non-TB) with minimal ambient interference and recorded in an identical environmental setup. Our findings support the feasibility of using a widely available recording device (smartphones) for a point-of-care cough-based TB screen and should be validated in different settings and patient populations. The smartphone-based model performed best in identifying participants with high GeneXpert grades or cavitary findings on lung disease, supporting a role for cough detection in identifying persons with pulmonary TB who are most infectious. Potential roles for this test include TB screening in peripheral levels of the health care system and as a public health intervention to screen congregate settings for interruption of transmission events.

## METHODS

### Nairobi dataset

Audio cough recordings of participants with pulmonary TB and control participants having non-TB–related cough (non-TB) were collected at the Centre for Respiratory Diseases Research (CRDR), Kenya Medical Research Institute (KEMRI), Nairobi, Kenya (Fig. 1). We recruited adult outpatients with TB from National Treatment Program clinics before starting anti-TB treatment. Pulmonary TB was diagnosed on the basis of a spontaneous sputum sample that was GeneXpert (MTB/RIF or Ultra) positive, subsequently confirmed by AFB culture. At the CRDR, sputum samples were decontaminated using *N*-acetyl-l-cysteine and sodium hydroxide and examined using fluorescence microscopy. If one or more AFB per equivalent of 100 immersion fields was observed, the slide was considered positive and graded on a scale of scanty, 1+, 2+, or 3+. After resuspension with phosphate buffer, equal sample volumes were used to perform mycobacterial culture and GeneXpert MTB/RIF or Ultra (Cepheid, Sunnyvale, CA). The GeneXpert assay assigns a semiquantitative category to positive tests for Mtb based on cycle threshold (Ct) values. GeneXpert MTB/RIF categories are high, medium, low, and very low; Ultra has an additional level, trace positive, the lowest level of detection. GeneXpert Ct values were assigned using the smallest Ct value from any of the probes targeting the rpoB gene; participants with Xpert Ultra trace-positive results (for whom rpoB probe Ct values were 0) were assigned a Ct value of 35 (the greatest Ct value for participants with an Xpert grade above trace was 30). Mycobacterial culture was performed using the MGIT Manual Mycobacterial Growth System (Becton-Dickinson, Franklin Lakes, NJ). Isolates were identified as Mtb using the Capilia TB Test Kit (TAUNS, Numazu, Japan). Sputum evaluations were performed on fresh samples. Per Kenya policy, patients with TB who were not known to be HIV positive underwent HIV testing. Participants with non-TB–related cough were adult outpatients recruited from the same National Treatment Program clinics or other chest clinics and were GeneXpert negative, had chest x-rays not typical for TB, and were determined to have cough due to conditions other than TB by study clinicians. Chest x-rays were evaluated for cavitary disease by a study investigator (D.J.H.). Various subcategories of semiquantitative GeneXpert and Sputum smear results were grouped into two categories—high and low. For GeneXpert, low category included trace, very low, and low results, and high category included medium and high semiquantitative readings. Similarly for sputum smear result, negative, scanty, and 1+ were classified as low and 2+ and 3+ results were categorized as high.

After obtaining informed consent (in English or Swahili), each participant sat in a quiet room for 2 hours with three recording devices recording continuous audio. The audio recording room was selected to be in a quieter location of the hospital grounds to minimize background noise interference, and participants were advised to minimize any phone conversation while the microphones were recording. The audio recording was annotated by humans (Supplementary Text and fig. S1) to mark coughs in the audio file. Three audio devices—a smartphone (Google Pixel 2), a low-cost boundary microphone (Codec), and a high-end condenser microphone (Yeti)—were used to record the audio at a sampling frequency of 44.1 kHz, 16 bit, and were placed on a table in front of the subject at a fixed distance. The latter two devices were plugged into a laptop computer for recording. Forced coughs from participants, where individuals were prompted to produce 10 coughs, were also recorded for a subset of participants. Raw audio data were uploaded to the Amazon Cloud Services S3 platform by the data collection team in Kenya. Along with audio recordings, we collected demographic (age, gender, smoking history, pulmonary health history, and HIV history) and clinical data (sputum analysis, chest x-ray, and blood samples) (Table 1). Study data were collected and managed using REDCap electronic data capture tools hosted at the University of Washington (*67*). The study was approved by the University of Washington (STUDY00009209) and KEMRI (KEMRI/SERU/CRDR/048/3988) Institutional Review Boards.

Audio files were annotated by human annotators at the University of Washington using Audacity software. Coughs with any background noise such as fan, door, speech, or any other respiratory sounds like a sneeze or clearing of nose/throat were discarded. In addition, cough audio files with any waveform distortion were removed from the dataset using amplitude-based thresholding (Supplementary Text). Each cough sound was processed to have a fixed length of 1 s, and recordings greater than a second were divided into multiple audio files. We fixed the audio length to 1 s similar to common audio classification models (*57*, *68*, *69*), which trade off between capturing majority of complete cough sounds versus enabling the future application to process audio in real time (*57*). Files with a length of less than 1 s were centered and padded with zeroes to make them 1 s long. Audio segments less than 0.1 s were discarded.

The passive cough dataset consists of 43,200 coughs, each 1 s long, from 149 subjects. After rejecting 4390 TB and 5169 non-TB coughs because of background noise and clipping, the total number of passive coughs in the Nairobi Cough dataset was 33,641 (TB: 23,191 and non-TB: 10,450) from all three recording devices and 149 subjects (TB: 103 and non-TB: 46). The forced cough set was reduced from 1619 to 1225 coughs [TB: 991 (42 subjects), non-TB: 234 (8 subjects)] after discarding 394 coughs because of clipping or background noise (Fig. 1B).

### Dataset—T1

We built a balanced subset having an equal number of TB/non-TB subjects (fig. S1A) to train and evaluate the binary classifier because the number of subjects in the non-TB group is lower than the TB group (Table 1). The balanced dataset consists of 45 non-TB subjects (1 subject was removed due to lack of sex information) and 45 TB subjects randomly sampled from the TB dataset. The sex distribution of TB (male: 27, female: 18) and non-TB (male: 27, female: 18) subjects was identical. We limited the maximum number of coughs per subject to 225 (the average number of coughs per subject in the dataset) to avoid signatures from any one subject dominating in training or evaluation. A dataset with 21,133 coughs (10,728 TB and 10,346 non-TB) was trained and tested with fivefold nested cross-validation (fig. S2A).

### Dataset—T2

T2 is the superset of T1 dataset and includes all non-TB (*n* = 1) and TB (*n* = 58) subjects not part of T1. It provides a fivefold unbalanced dataset (fig. S2B) with 33,641 passive coughs (TB: 23,191 and non-TB:10,450). Distribution of coughs based on the recording device, age, HIV infection, and smoking status is summarized in fig. S2B. This dataset is used as a test dataset, giving us performance metric for five folds using models trained on T1. Test folds are independent of training sets, for example, a model trained/validated on folds 2 to 5 of the T1 dataset are tested using fold 1 of T2.

### Dataset—T3

Forced coughs were used to evaluate the passive cough model trained using T1. A test set T3 was built for each fold such that coughs (passive or forced) from the same subject were not present in the training and the testing set simultaneously (fig. S2C and Table 1).

### Dataset—multiclass

Three subdatasets were built using passive coughs from the Nairobi dataset, each with three classes: non-TB (class 0), low TB presentation (class 1), and high TB presentation (class 2). Classes 1 and 2 were assigned by estimated Mtb bacillary burden (low or high for class 1 or 2, respectively) based on GeneXpert semiquantitative grade (class 1: trace, very low, and low; class 2: medium and high), sputum smear result (class 1: negative/scanty/1+; class 2: 2+/3+), or chest x-ray findings of cavitary disease absent (class 1) or present (class 2). Therefore, three different models based on GeneXpert (11,360 coughs, 83 subjects), sputum smear (14,578 coughs, 93 subjects), and chest x-ray (15,605, 102 subjects) were trained and evaluated using fivefold cross-validation. Each dataset is divided into five folds such that all the classes have equal number of subjects and similar gender distribution (fig. S5).

### Cough features

Time and frequency domain features were extracted from cough by converting the 1D time-series audio data to frequency domain using continuous wavelet transformation (*54*, *58*). The transformed data represented as images of cough scalograms were used to train and evaluate TBscreen classifier. Baseline models were trained and evaluated using mel-spectrograms features generated from the cough audio.

Scalograms for each second log audio clips were extracted using complex Morlet transformation (*54*). We selected the wavelet as the shape of the complex Morlet mother wavelet, a sine wave tapered by a Gaussian, resembles the shape of an audio waveform. After manual tuning, mother wavelet with bandwidth of 1.5 and center frequency of 1 was selected for our application and amplitudes were converted to log scale. To reduce redundancy, hundred scales (inversely related to frequency) with logarithmic spacing were selected in the frequency range of 10 to 16 kHz, resulting in a 2D scalogram of size 100 × 44,100. Boundary effects were mitigated by masking ${\sqrt{2}}^{*}\text{Scales}$ samples at each scale (*60*). Next, to reduce the dimensionality, instead of feeding the scalogram directly, resized and scaled color image (Python’s matplotlib) of the scalogram with amplitudes in logarithmic scale (448 × 224 × 3, PNG) was fed to the model. To generate images, different frequency ranges of wavelets were selected from 100 × 44,100 scalogram array to understand impact of frequency. Furthermore, scalograms were additionally generated with audio down-sampled to 8 kHz to compare its impact on modeling. Mel-spectrogram features for the ResNet18 baseline model were extracted from a second-long audio samples using PyTorch torchaudio’s mel-spectrogram transformation (window length: 0.025 s, hop length: 0.01 s, audio sampling rate: 44.1 kHz) with amplitudes in log scale. Inputs for VGGish were generated using the preprocessing pipeline proposed in the publication (window length: 0.025 s, hop length: 0.01 s, audio sampling rate: 16 kHz) (*57*). Further details for generating cough features are summarized in Supplementary Text and fig. S3 (A to F).

### TB—binary classification model architecture

ImageNet pretrained ResNet18 (*53*) was used to train the scalogram and log mel spectrogram based model. It has four residual convolutional blocks called the feature layers, followed by an adaptive average pooling layer and a final dense layer termed as the classification layer. The original model architecture was modified by replacing the classification layer (a single dense layer) with two dense layers. ReLu activation was used between the two dense layers and sigmoid activation for the final layer (Fig. 3B and table S3). The second baseline model was built using pretrained VGGish and modified to include an additional dense layer with sigmoid activation. Model architecture for baseline models is summarized in tables S5 and S6.

### TB—multiclass classification model architecture

This model was similar to the ResNet18-based binary classifier, apart from the output layer that had three classes instead of one (table S4). The input feature set consists of scalograms in the frequency range of 10 to 4 kHz from audio data sampled at 44.1 kHz.

### Training and evaluation

We used transfer learning by leveraging the image classification model ResNet18, pretrained with millions of images (*70*) to train our TB versus non-TB binary classifier. The scalogram image is different from the ImageNet dataset, which has been used to train the ResNet18, requiring us to fine-tune both the feature layer along with training the classification layers of the model. We used binary cross-entropy loss with the Adam optimizer to train the all-binary models. The model hyperparameters—learning rate for feature/classification layers, learning rate scheduler, and batch size—were tuned to adjust the model performance. Inputs to the model were adjusted with ImageNet-based default mean and SD for ResNet18. The model was trained for at least 20 epochs, after which training and validation loss were monitored to stop the model training early. The model training was stopped when the training loss did not improve for 10 consecutive epochs or the validation loss increased for 10 continuous epochs. The knee point of the training curve, the point at which training stabilizes, was calculated with Python’s kneed library, and the model thereafter with the best validation accuracy was selected for each fold (Supplementary Text and fig. S4). The baseline ResNet18 model was similarly trained using ImageNet’s pretrained weights and fine-tuned. VGGish model (*57*) was also initialized with pretrained weights, initially trained on Audioset, and further fine-tuned.

The classifiers were trained and tested using fivefold, nested, cross-validation with the balanced dataset divided into subject-independent five folds and having an equal number of TB and non-TB subjects. Gender distribution for TB and non-TB subjects was kept identical in each fold (Fig. 4C). In each of the five iterations, the model was tested on one of the folds while trained and tuned using the rest of the four folds (Fig. 3A). This was repeated five times so that each subfold acted as an independent test set, giving us five sets of model metrics. In each training, three of the four training folds were used for training, and one fold was set aside for validation to adjust the model hyperparameters. Because there were four folds in total for training and validation, we trained and validated four models and the best-performing model (fig. S4) was selected to evaluate the independent test set (Fig. 3). All models were trained with PyTorch using multiple GPUs (Nvidia RTX 2080 Ti, Quadro RTX 6000) part of the Hyak supercomputer system at the University of Washington.

Model performance was evaluated using ROC-AUC scores, sensitivity was calculated at 50% decision threshold, sensitivity was calculated at 70% specificity, and specificity was calculated at 50% decision threshold. Mean metric with SD across five folds is reported. In addition, supplemental tables are provided by aggregating results from all the five folds. Overall mean with 95% CI is estimated using stratified bootstrapping with *n* = 2000. The best-performing scalogram image–based binary model (highest ROC-AUC score) across different folds was trained with a learning rate of 0.000001 for feature layers, 0.00001 for the classification layer, a batch size of 32, and a scheduler that decreases the learning rate by 0.1 every 20 epochs. Training parameters for the best-performing models in various categories are summarized in table S2.

In training multiclass model, we used cross entropy loss with SoftMax activation for the output layer. The model was trained/validated using four of the five folds and evaluated on the one left-out fold. Performance was measured using overall accuracy, and class-specific metrics were calculated from the confusion matrix (table S2).

### Statistical analysis

The Mann-Whitney *U* test was used to assess statistical significance without any assumption of normality in the dataset. We assessed associations between cough frequency and TB characteristics using multivariate generalized linear regression models in which we included predictor variables (age, sex, HIV status, GeneXpert grade, GeneXpert Ct values, AFB smear grade, and chest x-ray cavitation) with *P* values of <0.20 in bivariate analyses. Using the “nest_reg_” command in Stata, models were compared using likelihood ratio tests. CIs were estimated using bootstrapping with *n* = 2000 iterations. Differences between ROC curves were tested for significance using DeLong’s test. Statistical tests were two-sided with α = 0.05, with only cough counts evaluated using greater hypothesis for TB cough. Analyses were performed using Stata 14 (StataCorp, College Station, TX), R: A Language and Environment for Statistical Computing, and Python.
