# Supplementary material for: TBscreen: A passive cough classifier for tuberculosis screening with a controlled dataset
Source: Sci Adv. 2024 Jan 3;10(1):eadi0282. doi: 10.1126/sciadv.adi0282 (PMC10776005; doi:10.1126/sciadv.adi0282)
Supplement: Supplementary file 1 — Supplementary Text Figs. S1 to S7 Tables S1 to S16 [file sciadv.adi0282_sm.pdf]

Science Advances
AAAS

Supplementary Materials for

TBscreen: A passive cough classifier for tuberculosis screening with a controlled dataset

Manuja Sharma *et al.*

Corresponding author: Manuja Sharma, manuja21@uw.edu

Sci. Adv. **10**, eadi0282 (2024)
DOI: 10.1126/sciadv.adi0282

This PDF file includes:

Supplementary Text
Figs. S1 to S7
Tables S1 to S16

Supplementary Text: Cough segmentation, Audio features and Model characteristics

Cough annotation

Cough annotation is performed in Audacity software by human annotators (Fig. S1). Audio length less than a second is centered and padded with zero to adjust the audio length to one second. Audio length greater than a second is divided into multiple audio files. Audio files with high incidents of amplitudes over 0.99 are removed. High incidence is estimated using an empirically tested ratio. Following code snippet is used to remove audio files with saturation noise.

```
max_v = 0.99
if max(normalized_audio) >= max_v:
  if np.count_nonzero(audio >= max_v)/audio.size >=ratio:
                    #remove audio file

where ratio is set to 0.0001 empirically
```

Cough spectral features

Methodology for generation of various cough features is summarized below:

Scalogram generation for audio sampled at 44.1 KHz
Features are computed using Python's PyWavelets (pywt) library on audio sampled at 44.1 kHz, as shown in Fig. S3(A). The mother wavelet selected is the Complex Morlet wavelet with a bandwidth of 1.5 and a center frequency of 1. The resulting wavelet amplitudes are transformed to a logarithmic scale. These wavelets are generated across a frequency range spanning from 10 Hz to 16 kHz with intervals of 100 frequencies, as illustrated in Fig. S3 (B). This process produces a multidimensional array of size 100x44100. The master wavelet array is then employed to derive sub-arrays, each corresponding to specific frequency ranges, as detailed in Fig. S3(D). The resulting arrays for various frequency ranges are subsequently transformed into images, with axes and color bars disabled. The default color space is utilized for the analysis, and the influence of the color space selection has not been explored in this study.

Scalogram generation for audio sampled at 8 KHz
In order to investigate the algorithm's sensitivity to changes in the sampling rate, we performed down-sampling of the audio to 8 kHz. Subsequently, we built a wavelet spanning the frequency range from 10 Hz to 4000 Hz, as depicted in Fig. S3(C).

Mel Spectrogram for ResNet18 based model
The mel spectrogram is created by employing PyTorch's mel spectrogram function with specific parameters: a window length of 0.025 seconds, a hop length of 0.01 seconds, and an audio sampling rate of 44.1 kHz. This process results in an array of dimensions 64x101. Subsequently,

the amplitudes are logarithmically scaled and normalized through min-max scaling. The resulting mel spectrogram is then used as input for the model, as illustrated in Fig. S3(E).

Mel Spectrogram for VGGish

The mel spectrogram is generated using the default parameters recommended by the VGGish model, designed for audio classification with audio sampled at 16 kHz. This procedure yields an array of dimensions 64x96. This mel spectrogram is then fed as input to the model, as represented in Fig. S3(F).

Model selection

The model is trained for a minimum of 20 epochs, during which both the training and validation loss are closely monitored. The training process incorporates an early stopping mechanism, which comes into effect if either of the following conditions is met:

- Training loss fails to exhibit improvement for a continuous span of 10 epochs.
- Validation loss experiences continuous increments over 10 consecutive epochs.

To determine the knee point of the training curve, an estimation is made to identify the point at which the training process stabilizes. This knee point is crucial in understanding when the model training reaches a satisfactory level of convergence. Model for epoch greater than knee point having highest validation accuracy is selected for evaluation on the test set.

```python
# Python snippet to calculate knee point of training curve

from kneed import KneeLocator
kneedle = KneeLocator (epochs, training_loss, S=3.0,
                       curve='convex', direction='decreasing')
kneedle.plot_knee()
```

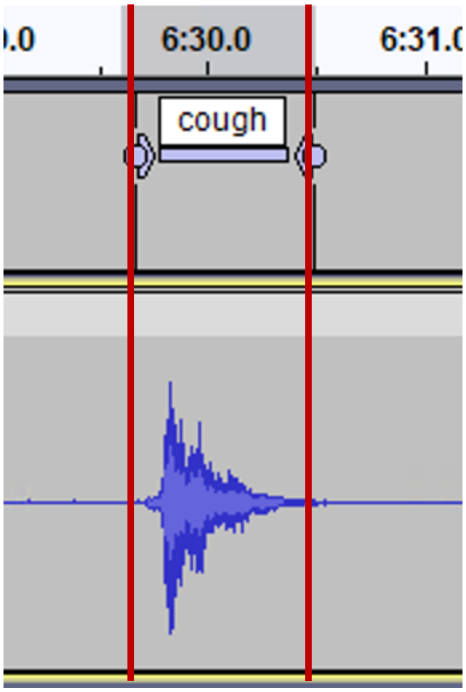

Fig. S1. Cough Annotation in Audacity

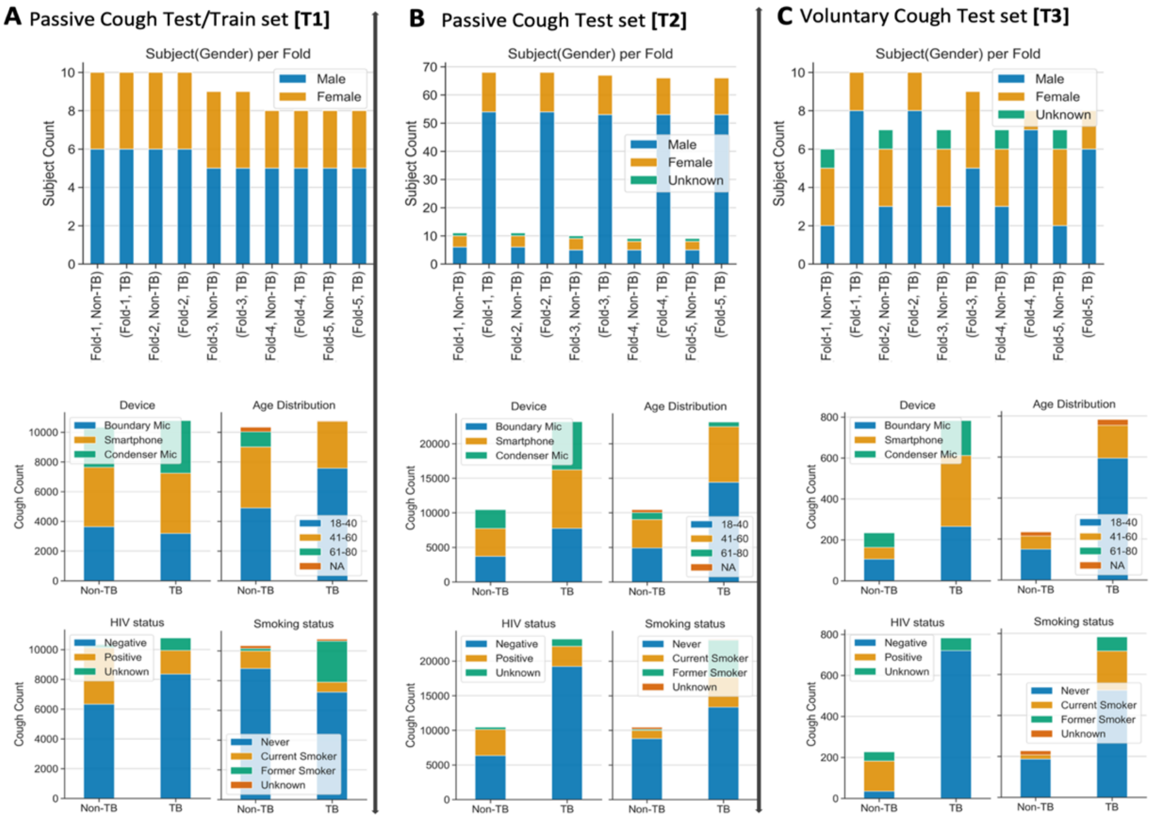

Fig. S2. Datasets used for training and testing of passive and voluntary binary cough classifier. (a) T1 Dataset: it has 5 subject-independent (unique subjects) folds with equal number of TB (n=45) and non-TB (n=45) subjects and identical gender distribution for both the classes. Distribution of cough in T1 with respect to recording devices, age, HIV status and smoking status is summarized. (b) T2 Dataset: an unbalanced test set consisting of coughs from all TB subjects (n=103) and all non-TB subjects (N=46) coughs in the dataset. T1 folds are extended to include all non-training data in the dataset, each fold is constructed such that there is no overlap of subjects in training and testing data. Distribution of cough in T2 w.r.t to recording devices, age, HIV status and smoking status is depicted. (c) T3 Dataset: this dataset contains voluntary coughs from TB (N=29) and non-TB (N=8) subjects, each fold is constructed such that there is no overlap of subjects in training and testing data. Distribution of cough in T3 with regards to to recording devices, age, HIV status and smoking status is depicted.

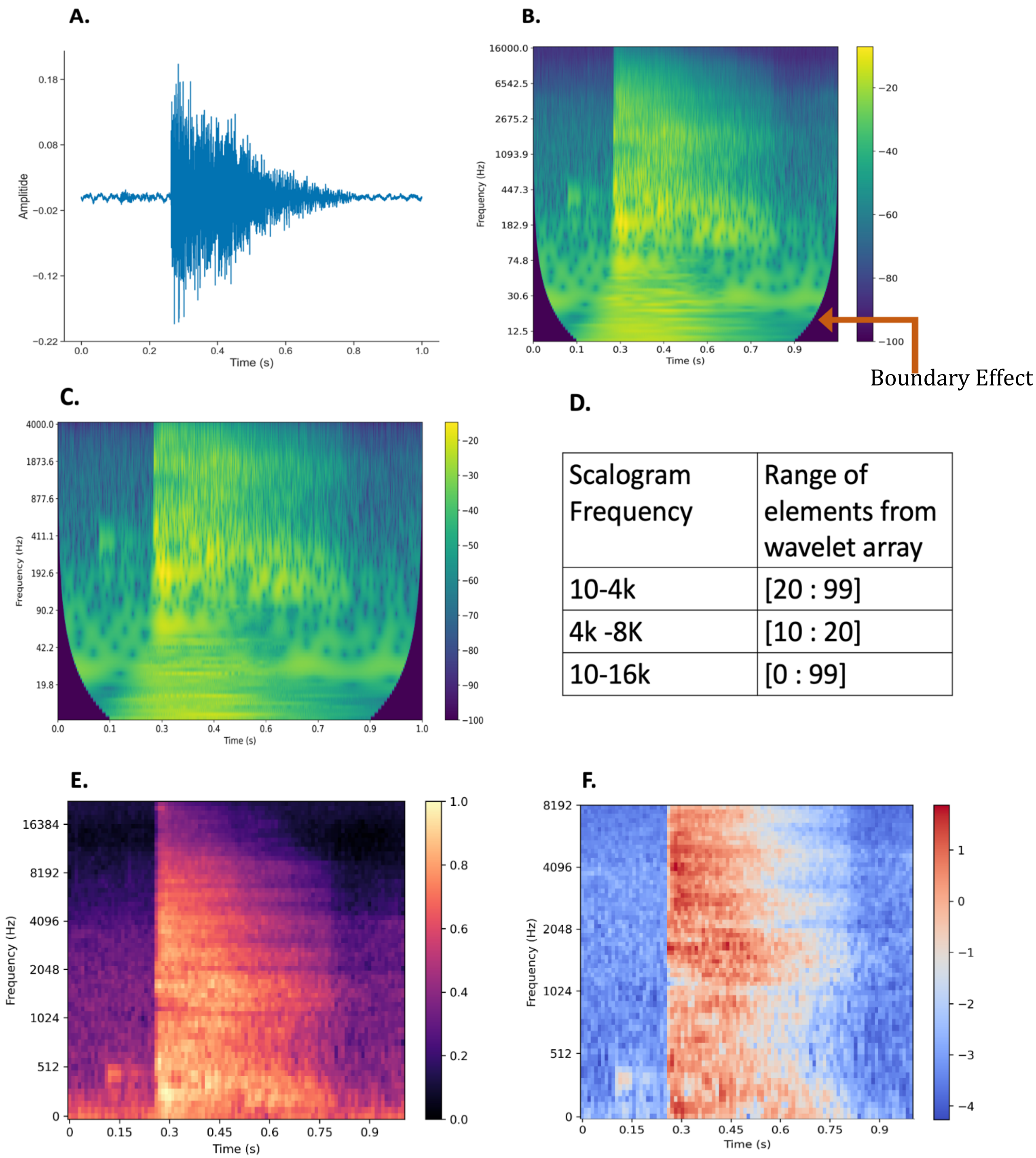

Scalogram Frequency	Range of elements from wavelet array
10-4k	[20 : 99]
4k -8K	[10 : 20]
10-16k	[0 : 99]

Fig. S3. Various audio features used in cough modeling. (A) Sound file captured using smartphone (B) Scalogram generated for audio sampled at 44.1KHz in the range of 10-16KHz with boundary effect data masked. The image without the color bar and axis label is sent as input to the model. (C) Scalogram generated for audio sampled at 8KHz in the range of 10-4KHz with boundary effect data masked (D) Range of array elements to represent different frequency range from wavelet generated using wavelet (100,10,16000,1.0/sr) where sr = 44.1KHz (E) Mel Spectrogram generated using Pytorch's inbuild mel spectrogram used as input to ResNet18 baseline model (F) Mel Spectrogram generated using VGGish parameters as input to VGGish baseline model.

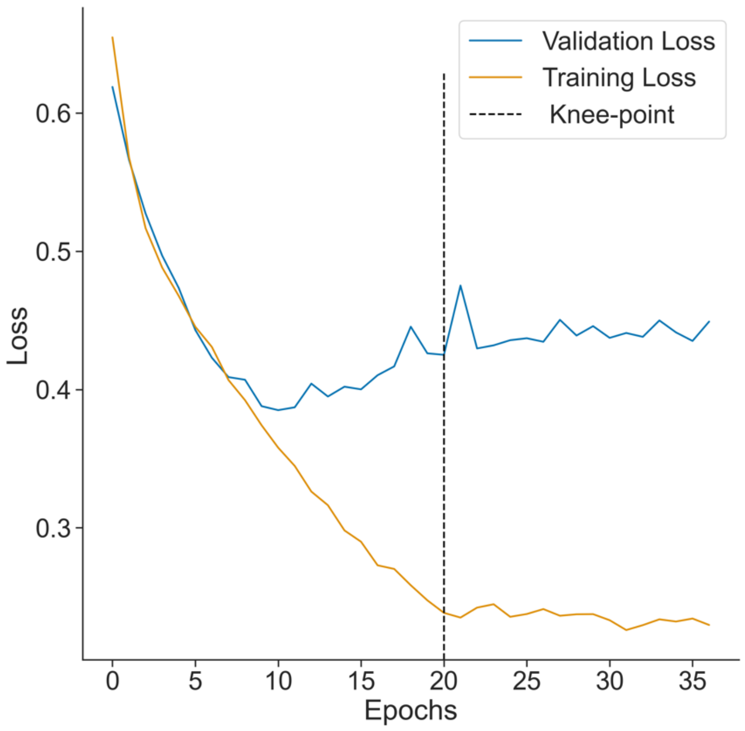

Fig. S4. Loss over epochs. Training and validation curve of model trained using scalograms generated at 44.1KHz, between 10-4KHz using data from all devices. Knee point is selected as 20 and models at epochs greater than 20 is selected for validation accuracy.

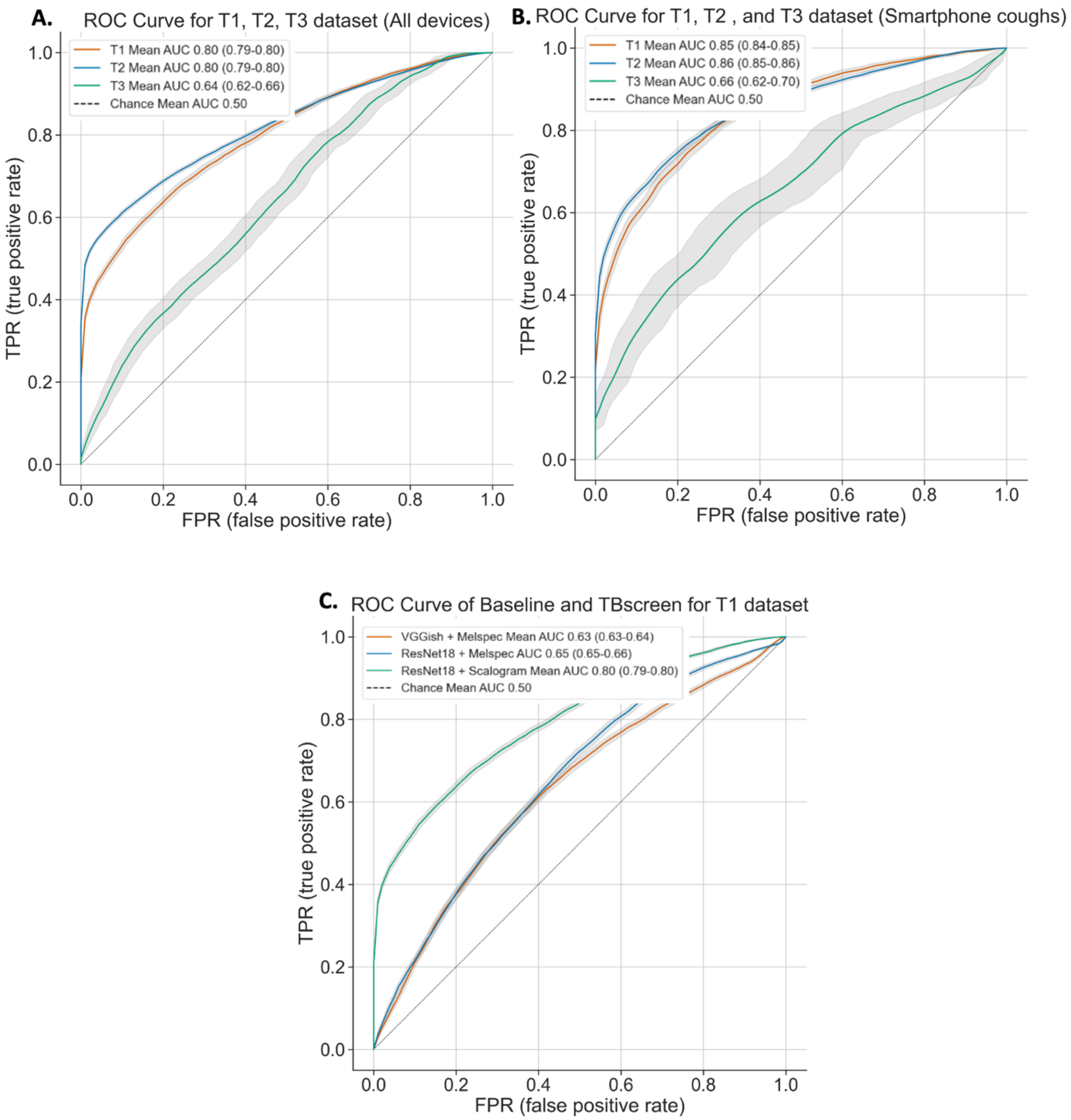

Fig. S5. Passive Binary Cough ROC plot with Confidence Interval. (a) ROC-curve with 95 % Confidence Interval after aggregating results from all folds. Models were trained using coughs from all devices and evaluated on T1: subject balanced passive cough dataset (w.r.t. to gender and number of subjects) and used for 5-fold training and testing of the classifier; T2: expanded T1 consisting of all non-TB subjects and TB cough data not included for training the 5-fold classifier; T3: a voluntary cough dataset consisting of coughs from TB and non-TB subjects. ROC curve with standard deviation for s second model trained on and validated on coughs from smartphone is also represented (b) ROC-curve with 95 % Confidence Interval after aggregating results for model trained using coughs from smartphone and evaluated on T1, T2, T3 (c) Comparison of ROC curve of the binary cough classifier trained using scalogram images of cough and baseline cough models trained on mel-spectrogram features with confidence interval. Results from all folds are aggregated and statistics estimated using bootstrapping (n =2000).

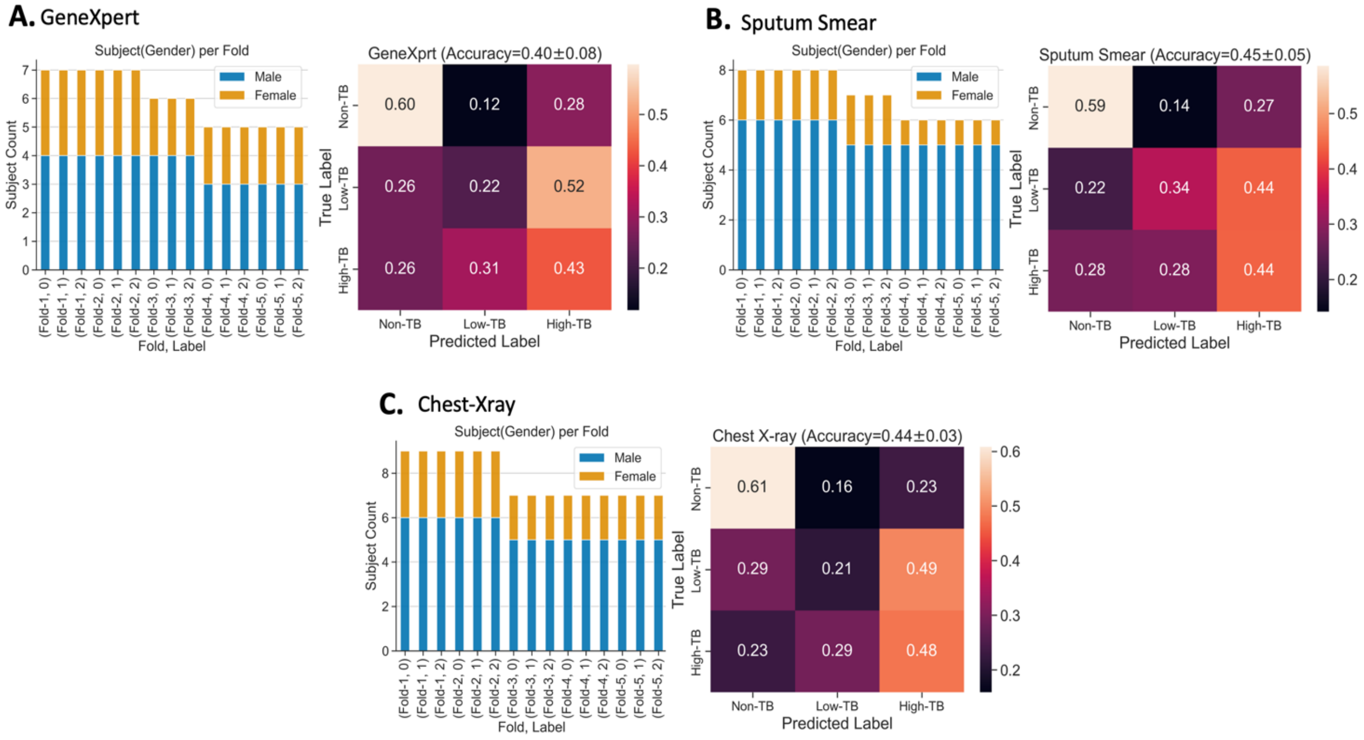

Fig. S6. Dataset and performance for multi-class classifier. Model using (a) GeneXpert levels, (b) sputum smear result, or (c) chest X-ray. Multi-Class normalized confusion matrix for four different types of classification is presented along with the subject/gender distribution in the 5-fold cross validation dataset. The confusion matrix summarizes classification results from all five folds.

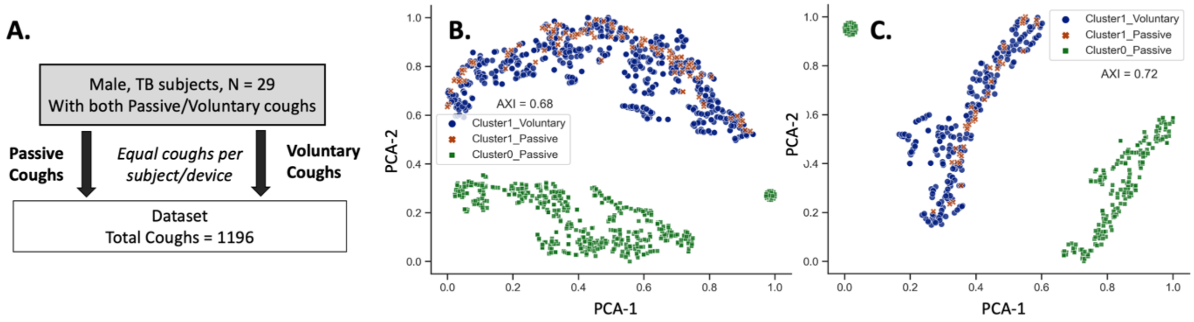

Fig. S7. Forced versus passive cough analysis. (a) We compared the distribution of features from the two types of coughs to analyze whether have similar properties. We then selected TB, male subjects, and equal number of coughs from each recording device to reduce variability in data, giving us a dataset of 1196 coughs from 29 male, TB subjects. (b) Coughs were converted to scalogram images, normalized, flattened (3, 448*224), and then reduced using PCA (variance of 0.98). The data were fitted using K-Means clustering with n=2 and clustering accuracy was measured using an adjusted random index (AXI). The PCA features were plotted using 2-D tsne to visualize the k-mean clusters. We used K-Means clustering using passive and voluntary coughs from same subjects. The clustering score, AXI is 68% indicating that the characteristics of voluntary coughs do differ from passive cough when analyzing coughs on all devices. The clusters are visualized using t-sne showing two clear clusters; cluster 0 only consists of passive coughs while cluster 1 is a mixture of some passive coughs and all of the voluntary coughs. (c) Clustering on smartphone: With clustering of a subset of this with coughs only from one recording device (smartphone), the clustering accuracy improves further to 72%. The results indicate that distribution of cough frequency features for voluntary and passive coughs differ forming almost separate clusters.

Variable	Coeff	Standard Error	z	P>z	95% Confidence Interval	Number of Observations
Age	-0.32	0.93	-0.34	0.73	-2.14, 1.51	103
Gender	-37.57	24.41	-1.54	0.12	-85.4, 10.3	103
HIV Infection	-3.31	34.58	-0.1	0.92	-71.1, 64.5	99
GeneXpert Semiquantitative grading	22.80	9.69	2.35	0.02	3.8, 41.8	102
Sputum smear	17.64	10.83	1.63	0.10	-3.6, 38.9	96
Cavity Chest X-ray	38.69	21.81	1.77	0.08	-4.1, 81.4	103

Table S1. Linear regression model of clinical variables and cough counts. A linear regression model was used to examine whether the indicated variables were associated with cough counts. The relationship between GeneXpert semi-quantitative scaling (1-5) and cough count was significant, for every increase in test level, cough count increases by 22.

Model	Total coughs	Coughs per fold	Batch Size	Learning Rate	Epochs	Schedular	Loss Function	Optimizer	Evaluation Metric
**TBscreen (All devices)**	21133	4226±670	32	Feature layers: 2e-05 Classification Layers: 1e-06	22	Pytorch's function: StepLR (optimizer, step_size=20, gamma=0.1)	Pytorch's function: BCEWithLogitsLoss()	Adam	Weighted ROC AUC score<br>Sensitivity at 50% decision threshold<br>Specificity at 50% decision threshold<br>Sensitivity at 70% specificity
**TBscreen (Smartphone)**	8054	1610±420	32	Feature layers: 2e-05 Classification Layers: 1e-06	38				
**TBscreen (Boundary Microphone)**	6255	1251±206	32	Feature layers: 2e-05 Classification Layers: 1e-06	35				
**TBscreen (Condenser Microphone)**	6824	1364±143	32	Feature layers: 2e-05 Classification Layers: 1e-06	33				
**Baseline (ResNet18)**	21133	4226±670	32	Feature layers: 2e-05 Classification Layers: 1e-06	25				
**Baseline (VGGish)**	21133	4226±670	32	Feature layers:0.00001 Classification Layers:0.0001	10				
**GeneXprt Multiclass Model**	11360	2272±589	32	Feature layers:0.00001 Classification Layers:0.0001	36		Pytorch's function: CrossEntropyLoss()		Accuracy = Total Correct Prediction/ Total predictions<br>Per class sensitivity = True Positive / (True Positive + False Negative)
**Sputum smear Multiclass Model**	14578	2915 ±731	32	Feature layers:0.00001 Classification Layers:0.0001	26				
**Chest X-ray Multiclass Model**	15605	3121 ± 255	32	Feature layers:0.00001 Classification Layers:0.0001	24				

Table S2: Model Training Parameters. Various training parameters for best performing models

Table S3: Binary TBscreen Model Architecture. ResNet18 based binary classifier with scalogram image of size [3*448*224] as input.

Layer (type)	Output Shape	Param #
Conv2d-1	[-1, 64, 224, 112]	9,408
BatchNorm2d-2	[-1, 64, 224, 112]	128
ReLU-3	[-1, 64, 224, 112]	0
MaxPool2d-4	[-1, 64, 112, 56]	0
Conv2d-5	[-1, 64, 112, 56]	36,864
BatchNorm2d-6	[-1, 64, 112, 56]	128
ReLU-7	[-1, 64, 112, 56]	0
Conv2d-8	[-1, 64, 112, 56]	36,864
BatchNorm2d-9	[-1, 64, 112, 56]	128
ReLU-10	[-1, 64, 112, 56]	0
BasicBlock-11	[-1, 64, 112, 56]	0
Conv2d-12	[-1, 64, 112, 56]	36,864
BatchNorm2d-13	[-1, 64, 112, 56]	128
ReLU-14	[-1, 64, 112, 56]	0
Conv2d-15	[-1, 64, 112, 56]	36,864
BatchNorm2d-16	[-1, 64, 112, 56]	128
ReLU-17	[-1, 64, 112, 56]	0
BasicBlock-18	[-1, 64, 112, 56]	0
Conv2d-19	[-1, 128, 56, 28]	73,728
BatchNorm2d-20	[-1, 128, 56, 28]	256
ReLU-21	[-1, 128, 56, 28]	0
Conv2d-22	[-1, 128, 56, 28]	147,456
BatchNorm2d-23	[-1, 128, 56, 28]	256
Conv2d-24	[-1, 128, 56, 28]	8,192
BatchNorm2d-25	[-1, 128, 56, 28]	256
ReLU-26	[-1, 128, 56, 28]	0
BasicBlock-27	[-1, 128, 56, 28]	0
Conv2d-28	[-1, 128, 56, 28]	147,456
BatchNorm2d-29	[-1, 128, 56, 28]	256
ReLU-30	[-1, 128, 56, 28]	0
Conv2d-31	[-1, 128, 56, 28]	147,456
BatchNorm2d-32	[-1, 128, 56, 28]	256
ReLU-33	[-1, 128, 56, 28]	0
BasicBlock-34	[-1, 128, 56, 28]	0
Conv2d-35	[-1, 256, 28, 14]	294,912
BatchNorm2d-36	[-1, 256, 28, 14]	512
ReLU-37	[-1, 256, 28, 14]	0
Conv2d-38	[-1, 256, 28, 14]	589,824
BatchNorm2d-39	[-1, 256, 28, 14]	512
Conv2d-40	[-1, 256, 28, 14]	32,768

```
     BatchNorm2d-41          [-1, 256, 28, 14]             512
            ReLU-42          [-1, 256, 28, 14]               0
      BasicBlock-43          [-1, 256, 28, 14]               0
          Conv2d-44          [-1, 256, 28, 14]         589,824
     BatchNorm2d-45          [-1, 256, 28, 14]             512
            ReLU-46          [-1, 256, 28, 14]               0
          Conv2d-47          [-1, 256, 28, 14]         589,824
     BatchNorm2d-48          [-1, 256, 28, 14]             512
            ReLU-49          [-1, 256, 28, 14]               0
      BasicBlock-50          [-1, 256, 28, 14]               0
          Conv2d-51           [-1, 512, 14, 7]       1,179,648
     BatchNorm2d-52           [-1, 512, 14, 7]           1,024
            ReLU-53           [-1, 512, 14, 7]               0
          Conv2d-54           [-1, 512, 14, 7]       2,359,296
     BatchNorm2d-55           [-1, 512, 14, 7]           1,024
          Conv2d-56           [-1, 512, 14, 7]         131,072
     BatchNorm2d-57           [-1, 512, 14, 7]           1,024
            ReLU-58           [-1, 512, 14, 7]               0
      BasicBlock-59           [-1, 512, 14, 7]               0
          Conv2d-60           [-1, 512, 14, 7]       2,359,296
     BatchNorm2d-61           [-1, 512, 14, 7]           1,024
            ReLU-62           [-1, 512, 14, 7]               0
          Conv2d-63           [-1, 512, 14, 7]       2,359,296
     BatchNorm2d-64           [-1, 512, 14, 7]           1,024
            ReLU-65           [-1, 512, 14, 7]               0
      BasicBlock-66           [-1, 512, 14, 7]               0
AdaptiveAvgPool2d-67            [-1, 512, 1, 1]               0
         Dropout-68                  [-1, 512]               0
          Linear-69                  [-1, 128]          65,664
            ReLU-70                  [-1, 128]               0
         Dropout-71                  [-1, 128]               0
          Linear-72                    [-1, 1]             129
================================================================
Total params: 11,242,305
Trainable params: 11,242,305
Non-trainable params: 0
----------------------------------------------------------------
Input size (MB): 1.15
Forward/backward pass size (MB): 125.57
Params size (MB): 42.89
Estimated Total Size (MB): 169.61
----------------------------------------------------------------
```

Table S4: Multiclass TBscreen Model Architecture. ResNet18 based multiclass classifier with scalogram image of size [3*448*224] as input.

Layer (type)	Output Shape	Param #
Conv2d-1	[-1, 64, 224, 112]	9,408
BatchNorm2d-2	[-1, 64, 224, 112]	128
ReLU-3	[-1, 64, 224, 112]	0
MaxPool2d-4	[-1, 64, 112, 56]	0
Conv2d-5	[-1, 64, 112, 56]	36,864
BatchNorm2d-6	[-1, 64, 112, 56]	128
ReLU-7	[-1, 64, 112, 56]	0
Conv2d-8	[-1, 64, 112, 56]	36,864
BatchNorm2d-9	[-1, 64, 112, 56]	128
ReLU-10	[-1, 64, 112, 56]	0
BasicBlock-11	[-1, 64, 112, 56]	0
Conv2d-12	[-1, 64, 112, 56]	36,864
BatchNorm2d-13	[-1, 64, 112, 56]	128
ReLU-14	[-1, 64, 112, 56]	0
Conv2d-15	[-1, 64, 112, 56]	36,864
BatchNorm2d-16	[-1, 64, 112, 56]	128
ReLU-17	[-1, 64, 112, 56]	0
BasicBlock-18	[-1, 64, 112, 56]	0
Conv2d-19	[-1, 128, 56, 28]	73,728
BatchNorm2d-20	[-1, 128, 56, 28]	256
ReLU-21	[-1, 128, 56, 28]	0
Conv2d-22	[-1, 128, 56, 28]	147,456
BatchNorm2d-23	[-1, 128, 56, 28]	256
Conv2d-24	[-1, 128, 56, 28]	8,192
BatchNorm2d-25	[-1, 128, 56, 28]	256
ReLU-26	[-1, 128, 56, 28]	0
BasicBlock-27	[-1, 128, 56, 28]	0
Conv2d-28	[-1, 128, 56, 28]	147,456
BatchNorm2d-29	[-1, 128, 56, 28]	256
ReLU-30	[-1, 128, 56, 28]	0
Conv2d-31	[-1, 128, 56, 28]	147,456
BatchNorm2d-32	[-1, 128, 56, 28]	256
ReLU-33	[-1, 128, 56, 28]	0
BasicBlock-34	[-1, 128, 56, 28]	0
Conv2d-35	[-1, 256, 28, 14]	294,912
BatchNorm2d-36	[-1, 256, 28, 14]	512
ReLU-37	[-1, 256, 28, 14]	0
Conv2d-38	[-1, 256, 28, 14]	589,824
BatchNorm2d-39	[-1, 256, 28, 14]	512
Conv2d-40	[-1, 256, 28, 14]	32,768

```
     BatchNorm2d-41          [-1, 256, 28, 14]             512
            ReLU-42          [-1, 256, 28, 14]               0
      BasicBlock-43          [-1, 256, 28, 14]               0
          Conv2d-44          [-1, 256, 28, 14]         589,824
     BatchNorm2d-45          [-1, 256, 28, 14]             512
            ReLU-46          [-1, 256, 28, 14]               0
          Conv2d-47          [-1, 256, 28, 14]         589,824
     BatchNorm2d-48          [-1, 256, 28, 14]             512
            ReLU-49          [-1, 256, 28, 14]               0
      BasicBlock-50          [-1, 256, 28, 14]               0
          Conv2d-51           [-1, 512, 14, 7]       1,179,648
     BatchNorm2d-52           [-1, 512, 14, 7]           1,024
            ReLU-53           [-1, 512, 14, 7]               0
          Conv2d-54           [-1, 512, 14, 7]       2,359,296
     BatchNorm2d-55           [-1, 512, 14, 7]           1,024
          Conv2d-56           [-1, 512, 14, 7]         131,072
     BatchNorm2d-57           [-1, 512, 14, 7]           1,024
            ReLU-58           [-1, 512, 14, 7]               0
      BasicBlock-59           [-1, 512, 14, 7]               0
          Conv2d-60           [-1, 512, 14, 7]       2,359,296
     BatchNorm2d-61           [-1, 512, 14, 7]           1,024
            ReLU-62           [-1, 512, 14, 7]               0
          Conv2d-63           [-1, 512, 14, 7]       2,359,296
     BatchNorm2d-64           [-1, 512, 14, 7]           1,024
            ReLU-65           [-1, 512, 14, 7]               0
      BasicBlock-66           [-1, 512, 14, 7]               0
AdaptiveAvgPool2d-67            [-1, 512, 1, 1]               0
         Dropout-68                  [-1, 512]               0
          Linear-69                  [-1, 128]          65,664
            ReLU-70                  [-1, 128]               0
         Dropout-71                  [-1, 128]               0
          Linear-72                    [-1, 3]             387
================================================================
Total params 11,242,563
Trainable params 11,242,563
Non-trainable params 0
----------------------------------------------------------------
Input size (MB) 1.15
Forwardbackward pass size (MB) 125.57
Params size (MB) 42.89
Estimated Total Size (MB) 169.61
----------------------------------------------------------------
```

Table S5: Baseline ResNet18 Model Architecture. ResNet18 with Mel Spectrogram of size [1,64,101] as input.

Layer (type)	Output Shape	Param #
Conv2d-1	[-1, 64, 32, 51]	3,136
BatchNorm2d-2	[-1, 64, 32, 51]	128
ReLU-3	[-1, 64, 32, 51]	0
MaxPool2d-4	[-1, 64, 16, 26]	0
Conv2d-5	[-1, 64, 16, 26]	36,864
BatchNorm2d-6	[-1, 64, 16, 26]	128
ReLU-7	[-1, 64, 16, 26]	0
Conv2d-8	[-1, 64, 16, 26]	36,864
BatchNorm2d-9	[-1, 64, 16, 26]	128
ReLU-10	[-1, 64, 16, 26]	0
BasicBlock-11	[-1, 64, 16, 26]	0
Conv2d-12	[-1, 64, 16, 26]	36,864
BatchNorm2d-13	[-1, 64, 16, 26]	128
ReLU-14	[-1, 64, 16, 26]	0
Conv2d-15	[-1, 64, 16, 26]	36,864
BatchNorm2d-16	[-1, 64, 16, 26]	128
ReLU-17	[-1, 64, 16, 26]	0
BasicBlock-18	[-1, 64, 16, 26]	0
Conv2d-19	[-1, 128, 8, 13]	73,728
BatchNorm2d-20	[-1, 128, 8, 13]	256
ReLU-21	[-1, 128, 8, 13]	0
Conv2d-22	[-1, 128, 8, 13]	147,456
BatchNorm2d-23	[-1, 128, 8, 13]	256
Conv2d-24	[-1, 128, 8, 13]	8,192
BatchNorm2d-25	[-1, 128, 8, 13]	256
ReLU-26	[-1, 128, 8, 13]	0
BasicBlock-27	[-1, 128, 8, 13]	0
Conv2d-28	[-1, 128, 8, 13]	147,456
BatchNorm2d-29	[-1, 128, 8, 13]	256
ReLU-30	[-1, 128, 8, 13]	0
Conv2d-31	[-1, 128, 8, 13]	147,456
BatchNorm2d-32	[-1, 128, 8, 13]	256
ReLU-33	[-1, 128, 8, 13]	0
BasicBlock-34	[-1, 128, 8, 13]	0
Conv2d-35	[-1, 256, 4, 7]	294,912
BatchNorm2d-36	[-1, 256, 4, 7]	512
ReLU-37	[-1, 256, 4, 7]	0
Conv2d-38	[-1, 256, 4, 7]	589,824
BatchNorm2d-39	[-1, 256, 4, 7]	512
Conv2d-40	[-1, 256, 4, 7]	32,768

```
     BatchNorm2d-41            [-1, 256, 4, 7]             512
            ReLU-42            [-1, 256, 4, 7]               0
      BasicBlock-43            [-1, 256, 4, 7]               0
          Conv2d-44            [-1, 256, 4, 7]         589,824
     BatchNorm2d-45            [-1, 256, 4, 7]             512
            ReLU-46            [-1, 256, 4, 7]               0
          Conv2d-47            [-1, 256, 4, 7]         589,824
     BatchNorm2d-48            [-1, 256, 4, 7]             512
            ReLU-49            [-1, 256, 4, 7]               0
      BasicBlock-50            [-1, 256, 4, 7]               0
          Conv2d-51            [-1, 512, 2, 4]       1,179,648
     BatchNorm2d-52            [-1, 512, 2, 4]           1,024
            ReLU-53            [-1, 512, 2, 4]               0
          Conv2d-54            [-1, 512, 2, 4]       2,359,296
     BatchNorm2d-55            [-1, 512, 2, 4]           1,024
          Conv2d-56            [-1, 512, 2, 4]         131,072
     BatchNorm2d-57            [-1, 512, 2, 4]           1,024
            ReLU-58            [-1, 512, 2, 4]               0
      BasicBlock-59            [-1, 512, 2, 4]               0
          Conv2d-60            [-1, 512, 2, 4]       2,359,296
     BatchNorm2d-61            [-1, 512, 2, 4]           1,024
            ReLU-62            [-1, 512, 2, 4]               0
          Conv2d-63            [-1, 512, 2, 4]       2,359,296
     BatchNorm2d-64            [-1, 512, 2, 4]           1,024
            ReLU-65            [-1, 512, 2, 4]               0
      BasicBlock-66            [-1, 512, 2, 4]               0
AdaptiveAvgPool2d-67           [-1, 512, 1, 1]               0
         Dropout-68                  [-1, 512]               0
          Linear-69                  [-1, 128]          65,664
            ReLU-70                  [-1, 128]               0
         Dropout-71                  [-1, 128]               0
          Linear-72                    [-1, 1]             129
================================================================
Total params: 11,236,033
Trainable params: 11,236,033
Non-trainable params: 0
----------------------------------------------------------------
Input size (MB): 0.02
Forward/backward pass size (MB): 8.45
Params size (MB): 42.86
Estimated Total Size (MB): 51.33
----------------------------------------------------------------
```

Table S6: Baseline VGGish Model Architecture. Model with Mel Spectrogram of size [1,64,96] as input

Layer (type)	Output Shape	Param #
Conv2d-1	[-1, 64, 64, 96]	640
ReLU-2	[-1, 64, 64, 96]	0
MaxPool2d-3	[-1, 64, 32, 48]	0
Conv2d-4	[-1, 128, 32, 48]	73,856
ReLU-5	[-1, 128, 32, 48]	0
MaxPool2d-6	[-1, 128, 16, 24]	0
Conv2d-7	[-1, 256, 16, 24]	295,168
ReLU-8	[-1, 256, 16, 24]	0
Conv2d-9	[-1, 256, 16, 24]	590,080
ReLU-10	[-1, 256, 16, 24]	0
MaxPool2d-11	[-1, 256, 8, 12]	0
Conv2d-12	[-1, 512, 8, 12]	1,180,160
ReLU-13	[-1, 512, 8, 12]	0
Conv2d-14	[-1, 512, 8, 12]	2,359,808
ReLU-15	[-1, 512, 8, 12]	0
MaxPool2d-16	[-1, 512, 4, 6]	0
Linear-17	[-1, 4096]	50,335,744
ReLU-18	[-1, 4096]	0
Linear-19	[-1, 4096]	16,781,312
ReLU-20	[-1, 4096]	0
Linear-21	[-1, 128]	524,416
ReLU-22	[-1, 128]	0
VGGish-23	[-1, 128]	0
Linear-24	[-1, 1]	129
addlayer-25	[-1, 1]	0

Total params: 72,141,313
Trainable params: 72,141,313
Non-trainable params: 0

Input size (MB): 0.02
Forward/backward pass size (MB): 15.03
Params size (MB): 275.20
Estimated Total Size (MB): 290.25

	Model Training Parameters	Test Set	ROC-AUC score (95% CI)	Sensitivity (95% CI)	Specificity (95% CI)	Sensitivity @70% Specificity (95% CI)
TBscreen	Device: All,<br><br>Scalogram:<br>10 Hz – 4 KHz,<br>Sampling rate: 44.1 KHz	T1: Subject Balanced CV	0.80 (0.79-0.80)	0.72 (0.71-0.73)	0.70 (0.69-0.72)	0.71 (0.71-0.73)
		T2: Expanded T1, Unbalanced set	0.80 (0.79-0.80)	0.72 (0.71-0.72)	0.70 (0.69-0.72)	0.72 (0.71-0.73)
		T3: Voluntary cough, Unbalanced set	0.64 (0.62-0.66)	0.35 (0.31-0.38)	0.83 (0.79-0.85)	0.46 (0.43-0.50)
TBscreen<br><br>Trained/ Evaluated on coughs from Smartphone	Device: Smartphone,<br><br>Scalogram:<br>10 Hz – 4 KHz,<br>Sampling rate: 44.1 KHz	T1 subset: Subject Balanced CV	0.85 (0.84-0.85)	0.79 (0.7-0.81)	0.73 (0.71-0.74)	0.81 (0.79-0.83)
		T2 subset: Expanded T1, Unbalanced set	0.86 (0.85-0.86)	0.81 (0.80-0.82)	0.73 (0.71-0.75)	0.83 (0.82-0.84)
		T3 subset: Voluntary cough, Unbalanced set	0.66 (0.62-0.70)	0.20 (0.15-0.28)	0.96 (0.92-0.99)	0.54 (0.45-0.62)

Table S7. Performance across datasets with confidence interval: Average ROC-AUC score, sensitivity, and specificity along with 95% confidence interval using different training data (coughs from all devices vs coughs from smartphone) and test sets (T1, T2, T3). Two variations of the classifier are tested on three different test sets, T1: Subject balanced passive cough dataset (for gender and number of subjects) and used for 5-fold training and testing of the classifier; T2: Expanded T1 consisting of all non-TB subjects and TB cough data not included for training the 5-fold classifier; T3: a voluntary cough dataset consisting of coughs from TB and non-TB subjects. Results from all five folds are aggregated for each model and statistics estimated using bootstrapping (n =2000).

	Model Training Parameters	Test Set	ROC-AUC score (Average of 5-folds ± S.D. across folds)	Sensitivity (Average of 5-folds ± S.D. across folds)	Specificity (Average of 5-folds ± S.D. across folds)	Sensitivity @70% specificity (Average of 5-folds ± S.D. across folds)	Combined ROC-AUC score of 5 folds (Average of 5-folds with Confidence Interval)
TBscreen<br><br>Trained/Evaluated on coughs from Smartphone	Device: Smartphone,<br><br>Scalogram: 10 Hz – 4 KHz, Sampling rate: 44.1 KHz	T1 subset: Subject Balanced CV	0.83±0.11	0.76±0.12	0.74±0.10	0.76±0.20	0.85 (0.84-0.85)
		T2 subset: Expanded T1, Unbalanced set	0.86±0.03	0.80±0.03	0.74±0.10	0.83±0.05	0.86 (0.85-0.87)
		T3 subset: Voluntary cough, Unbalanced set	0.61±0.14	0.16±0.11	0.95±0.05	0.51±0.18	0.66 (0.62-0.70)
TBscreen<br><br>Trained/Evaluated on coughs from Boundary Microphone	Device: Boundary Microphone,<br><br>Scalogram: 10 Hz – 4 KHz, Sampling rate: 44.1 KHz	T1 subset: Subject Balanced CV	0.77±0.10	0.69±0.09	0.67±0.20	0.69±0.13	0.78 (0.77-0.78)
		T2 subset: Expanded T1, Unbalanced set	0.81±0.06	0.73±0.04	0.69±0.18	0.73±0.07	0.79 (0.78-0.80)
		T3 subset: Voluntary cough, Unbalanced set	0.61±0.08	0.44±0.13	0.68±0.11	0.47±0.13	0.62 (0.59-0.66)
TBscreen<br><br>Trained/Evaluated on coughs from Condenser Microphone	Device: Condenser Microphone,<br><br>Scalogram: 10 Hz – 4 KHz, Sampling rate: 44.1 KHz	T1 subset: Subject Balanced CV	0.73±0.14	0.65±0.19	0.65±0.13	0.62±0.22	0.73 (0.72-0.74)
		T2 subset: Expanded T1, Unbalanced set	0.80±0.04	0.75±0.06	0.65±0.12	0.73±0.06	0.74 (0.73-0.75)
		T3 subset: Voluntary cough, Unbalanced set	0.69±0.07	0.46±0.19	0.74±0.14	0.60±0.10	0.66 (0.62-0.70)

Table S8. Performance across datasets using different recording devices: Average ROC-AUC score, sensitivity, and specificity with standard deviation across 5-folds using different training data from different devices and test sets (T1, T2, T3). Device specific results are presented (Additional data for Table 2 in main text).

	Model Training Parameters	Test Set	ROC-AUC score (95% CI)	Sensitivity (95% CI)	Specificity (95% CI)	Sensitivity @70% specificity (95% CI)
TBscreen<br><br>Trained/Evaluated on coughs from Smartphone	Device: Smartphone, Scalogram: 10 Hz – 4 KHz, Sampling rate: 44.1 KHz	T1 subset: Subject Balanced CV	0.85 (0.84-0.85)	0.79 (0.7-0.81)	0.73 (0.71-0.74)	0.81 (0.79-0.83)
		T2 subset: Expanded T1, Unbalanced set	0.86 (0.85-0.86)	0.81 (0.80-0.82)	0.73 (0.71-0.75)	0.83 (0.82-0.84)
		T3 subset: Voluntary cough, Unbalanced set	0.66 (0.62-0.70)	0.20 (0.15-0.28)	0.96 (0.92-0.99)	0.54 (0.45-0.62)
TBscreen<br><br>Trained/Evaluated on coughs from Boundary Microphone	Device: Boundary Microphone, Scalogram: 10 Hz – 4 KHz, Sampling rate: 44.1 KHz	T1 subset: Subject Balanced CV	0.78 (0.77-0.79)	0.70 (0.69-0.72)	0.70 (0.67-0.73)	0.70 (0.68-0.72)
		T2 subset: Expanded T1, Unbalanced set	0.79 (0.78-0.80)	0.69 (0.68-0.70)	0.70 (0.68-0.72)	0.69 (0.68-0.70)
		T3 subset: Voluntary cough, Unbalanced set	0.62 (0.59-0.66)	0.47 (0.38-0.55)	0.70 (0.64-0.75)	0.46 (0.36-0.54)
TBscreen<br><br>Trained/Evaluated on coughs from Condenser Microphone	Device: Condenser Microphone, Scalogram: 10 Hz – 4 KHz, Sampling rate: 44.1 KHz	T1 subset: Subject Balanced CV	0.73 (0.72-0.74)	0.67 (0.65-0.69)	0.63 (0.60-0.66)	0.62 (0.60-0.64)
		T2 subset: Expanded T1, Unbalanced set	0.74 (0.73-0.75)	0.68 (0.66-0.69)	0.63 (0.60-0.66)	0.64 (0.62-0.65)
		T3 subset: Voluntary cough, Unbalanced set	0.66 (0.62-0.70)	0.47 (0.40-0.55)	0.76 (0.68-0.83)	0.54 (0.45-0.62)

Table S9. Performance across datasets using different recording devices along with confidence interval: Average ROC-AUC score, sensitivity, and specificity with 95 % confidence interval using different training data from different devices and test sets (T1, T2, T3). Device specific results are presented. Results from all five folds are aggregated for each model and statistics estimated using bootstrapping (n =2000).

	Model Training Parameters	Test Set	ROC-AUC score (95% CI)	Sensitivity (95% CI)	Specificity (95% CI)	Sensitivity @70% specificity (95% CI)
Frequency range of scalogram<br><br>Device: All, Sampling rate: 44.1 KHz	10 Hz – 4 KHz	T1: Subject Balanced CV	0.80 (0.79-0.80)	0.72 (0.71-0.73)	0.70 (0.69-0.72)	0.71 (0.71-0.73)
	4 KHz – 8 KHz	T1: Subject Balanced CV	0.75 (0.75-0.76)	0.68 (0.66-0.69)	0.68 (0.66-0.69)	0.66 (0.64-0.67)
	10 KHz – 16 KHz	T1: Subject Balanced CV	0.80 (0.79-0.80)	0.73 (0.72-0.74)	0.69 (0.67-0.70)	0.72 (0.71-0.73)
Sampling rate<br><br>Device: All	8 KHz Scalogram: 10 Hz – 4KHz	T1: Subject Balanced CV	0.63 (0.62-0.63)	0.64 (0.62-0.65)	0.54 (0.53-0.56)	0.47 (0.45-0.48)
	44.1 KHz Scalogram: 10 Hz – 4KHz	T1: Subject Balanced CV	0.80 (0.79-0.80)	0.72 (0.71-0.73)	0.70 (0.69-0.72)	0.71 (0.71-0.73)
Recording device<br><br>Scalogram: 10 Hz - 4KHz, Sampling rate: 44.1 KHz	Smartphone	T1 subset: Subject Balanced CV (Smartphone coughs)	0.85 (0.84-0.85)	0.79 (0.7-0.81)	0.73 (0.71-0.74)	0.81 (0.79-0.83)
		T1: Subject Balanced CV	0.80 (0.79-0.80)	0.77 (0.76-0.78)	0.63 (0.61-0.64)	0.72 (0.71-0.73)
	Boundary Mic.	T1 subset: Subject Balanced CV (Boundary Microphone coughs)	0.78 (0.77-0.79)	0.70 (0.69-0.72)	0.70 (0.67-0.73)	0.70 (0.68-0.72)
		T1: Subject Balanced CV	0.79 (0.79-0.80)	0.74 (0.73-0.75)	0.67 (0.66-0.69)	0.72 (0.71-0.73)
	Condenser Mic.	T1 subset: Subject Balanced CV (Condenser Microphone coughs)	0.73 (0.72-0.74)	0.67 (0.65-0.69)	0.63 (0.60-0.66)	0.62 (0.60-0.64)
		T1: Subject Balanced CV	0.69 (0.68-0.69)	0.68 (0.67-0.69)	0.51 (0.49-0.52)	0.53 (0.52-0.54)
Alternative Frequency Representation<br><br>Mel-spectrogram Device: All Baseline Model	Model: VGGish<br><br>Sampling rate: 16 KHz	T1: Subject Balanced CV	0.63 (0.63-0.64)	0.62 (0.61-0.63)	0.59 (0.58-0.60)	0.51 (0.49-0.52)
	Model: ResNet18<br><br>Sampling rate: 44.1 KHz	T1: Subject Balanced CV	0.65 (0.65-0.66)	0.64 (0.63-0.66)	0.57 (0.56-0.59)	0.50 (0.49-0.52)

Table S10. Various cough features and aggregated model performance with confidence interval: Average ROC-AUC score, sensitivity, and specificity with 95% confidence interval using different training inputs and performance evaluated on T1 dataset. Results from all five folds are aggregated for each model and statistics estimated using bootstrapping (n =2000).

	Category	Sub-Category	ROC-AUC score (95% CI)	Sensitivity (95% CI)	Specificity (95% CI)	Sensitivity @70% specificity (95% CI)
Overall Model<br><br>Device: Smartphone, Scalogram: 10 Hz – 4KHz, Sampling rate: 44.1 KHz	All inclusive	-	0.85 (0.84-0.85)	0.79 (0.7-0.81)	0.73 (0.71-0.74)	0.81 (0.79-0.83)
Demographic Bias	Gender	Male	0.89 (0.89-0.90)	0.88 (0.86-0.89)	0.72 (0.69-0.74)	0.89 (0.87-0.90)
		Female	0.76 (0.74-0.78)	0.60 (0.55-0.64)	0.75 (0.72-0.78)	0.66 (0.62-0.71)
	Age Group	[18,40]	0.85 (0.84-0.86)	0.82 (0.80-0.84)	0.68 (0.65-0.71)	0.81 (0.79-0.83)
		(40,60]	0.83 (0.81-0.84)	0.74 (0.71-0.78)	0.74 (0.71-0.77)	0.79 (0.76-0.81)
Clinical Bias	HIV History	No HIV history	0.84 (0.83-0.85)	0.78 (0.76-0.80)	0.72 (0.69-0.74)	0.79 (0.78-0.81)
		With HIV history	0.91 (0.90-0.93)	0.90 (0.87-0.93)	0.73 (0.68-0.80)	0.92 (0.89-0.94)
	Smoking History	No Smoking History	0.81 (0.80-0.83)	0.74 (0.72-0.76)	0.72 (0.70-0.75)	0.77 (0.74-0.79)
		With Smoking History	0.90 (0.89-0.91)	0.87 (0.84-0.90)	0.72 (0.68-0.77)	0.88 (0.86-0.91)
TB Presentations	GeneXpert	Low Bacterial Load	0.74 (0.73-0.76)	0.62 (0.59-0.66)	0.73 (0.70-0.76)	0.66 (0.62-0.69)
		High Bacterial Load	0.87 (0.86-0.88)	0.83 (0.82-0.85)	0.73 (0.71-0.75)	0.85 (0.84-0.87)
	Sputum Smear	Low Bacterial Load	0.85 (0.83-0.87)	0.79 (0.76-0.83)	0.73 (0.66-0.78)	0.81 (0.78-0.84)
		High Bacterial Load	0.86 (0.85-0.87)	0.82 (0.80-0.84)	0.73 (0.70-0.75)	0.84 (0.83-0.86)
	Lung Cavity	No Cavity	0.71 (0.69-0.72)	0.53 (0.50-0.57)	0.73 (0.70-0.75)	0.58 (0.54-0.62)
		With Cavity	0.89 (0.88-0.89)	0.86 (0.85-0.88)	0.73 (0.70-0.75)	0.88 (0.86-0.89)

Table S11. Smartphone classification and performance bias analysis of binary cough model with confidence interval: The classification results (mean with 95% confidence interval) are represented for different demographic, clinical and TB presentations in T1 dataset. Results from all five folds are aggregated for each model and statistics estimated using bootstrapping (n =2000).

	Model Training Parameters	Test Set	P - value
TBscreen<br><br>Trained/Evaluated on coughs from one recording device	T1<br>Scalogram:<br>10 Hz – 4 KHz,<br>Sampling rate:<br>44.1 KHz	Smartphone| Boundary Microphone	2e-14*
		Smartphone| Condenser Microphone	2e-16*
		Condenser Microphone | Boundary Microphone	4e-11*
TBscreen<br><br>Trained/Evaluated on coughs from one recording device	T2<br>Scalogram:<br>10 Hz – 4 KHz,<br>Sampling rate:<br>44.1 KHz	Smartphone| Boundary Microphone	2e-16*
		Smartphone| Condenser Microphone	2e-16*
		Condenser Microphone | Boundary Microphone	5e-14*
TBscreen<br><br>Trained/Evaluated on coughs from one recording device	T3<br>Scalogram:<br>10 Hz – 4 KHz,<br>Sampling rate:<br>44.1 KHz	Smartphone| Boundary Microphone	0.2
		Smartphone| Condenser Microphone	1
		Condenser Microphone | Boundary Microphone	0.2

Table S12. De-Long test results for performance across datasets using different recording devices: P<0.05 marked with * indicate significant difference between the ROC curves of models using Delong test.

**Subject Category**<br><br>Model: Smartphone coughs based, 10-4KHz, 44.1KHz	**No. of Subject**	**Mean accuracy (per subject)**	**95 % Confidence Interval**	**Mean accuracy (per cough)**
TB, Dataset T1	45	0.66	(0.56, 0.76)	0.76$\pm$0.12
TB, Dataset T2	103	0.68	(0.61, 0.75)	0.76$\pm$0.12
Non-TB, Dataset T1	45	0.78	(0.70, 0.86)	0.74$\pm$0.10

Table S13. TBscreen model accuracy per subject. Predictive accuracy of smartphone-based model per TB /non-TB subject using dataset T1 and T2.

TB Presentation	Sub-Category	No. of subjects	Accuracy	Sensitivity	Specificity
GeneXpert	Overall	90	0.40±0.09	-	-
	Class 0: Non-TB	30	0.69±0.05	0.58±0.23	0.74±0.11
	Class 1: GeneXpert 1-3	30	0.60±0.06	0.21±0.08	0.77±0.08
	Class 2: GeneXpert 4-5	30	0.51±0.11	0.42±0.17	0.57±0.15
Sputum Smear	Overall	117	0.45±0.05	-	-
	Class 0: Non-TB	39	0.70+0.10	0.58+0.05	0.76+0.13
	Class 1: Sputum smear 0-3	39	0.64+0.44	0.33+0.15	0.78+0.07
	Class 2: Sputum smear 4-5	39	0.57+0.04	0.45+0.13	0.64+0.03
Chest X-ray	Overall	105	0.44±0.03	-	-
	Class 0: Non-TB	35	0.70±0.06	0.62±0.07	0.74±0.05
	Class 1: TB, without cavity	35	0.60±0.05	0.23±0.08	0.78±0.06
	Class 2: TB, with cavity	35	0.58±0.04	0.49±0.11	0.65±0.11

Table S14. Multi-class model of TB presentation. Performance metrics of models based on GeneXpert, Sputum smear and Chest X-ray. The model included three distinct classes- non-TB (class-0), low-TB burden presentation (class-1) and high-TB burden presentation (class-2). The level of TB burden was either based on Mtb bacillary burden estimated using GeneXpert or sputum smear result (low vs high bacterial load); or on the presence (high)/absence (low) of lung cavities. Model accuracy along with sensitivity, specificity, and accuracy for each class is represented.

	Model Training Parameters	Test Set	P value
Frequency range of scalogram<br><br>Device: All,<br>Sampling rate: 44.1 KHz	10 Hz – 4 KHz | 10 KHz – 16 KHz	T1: Subject Balanced CV	0.8
	10 Hz – 4 KHz | 4 KHz – 8 KHz	T1: Subject Balanced CV	2e-16*
	4 KHz – 8 KHz| 10 KHz – 16 KHz	T1: Subject Balanced CV	2e-16*
Sampling rate<br><br>Device: All<br>Scalogram: 10 Hz – 4KHz	8 KHz| 44.1 KHz	T1: Subject Balanced CV	2e-16*
Recording device<br><br>Scalogram: 10 Hz -4KHz,<br>Sampling rate: 44.1 KHz	Smartphone | Boundary Mic.	T1 subset: Subject Balanced CV (Smartphone| Boundary Mic. coughs)	2e-14*
	Smartphone | Condenser Mic.	T1 subset: Subject Balanced CV (Smartphone| Condenser Mic. coughs)	2e-16*
	Boundary Mic.| Condenser Mic.	T1 subset: Subject Balanced CV (Boundary Mic. coughs | Condenser Mic. coughs)	4e-11*
Alternative Frequency Representation<br><br>Mel-spectrogram<br>Device: All<br>Baseline Model	VGGish | TBscreen	T1: Subject Balanced CV	2e-16*
	ResNet18 | TBscreen	T1: Subject Balanced CV	2e-16*
	VGGish | ResNet18	T1: Subject Balanced CV	6e-07*

Table S15. Statistical significance in comparing model performance: P<0.05 marked with * indicate significant difference between the models using Delong test.

	Category	Sub-Category	P-score	Coughs Control vs Case	Subjects Control vs Case
Demographic Bias	Gender	Male | Female	P = 2e-16*	(2930 vs. 2786) | (1068 vs. 1270)	(27 vs 27) | (17 vs 18)
	Age Group	[18,40] | (40,60]	P = 0.01*	(2069 vs. 2394) | (1068 vs. 1270)	(22 vs 22) | (17 vs 17)
Clinical Bias	HIV History	No HIV history | With HIV history	P = 8e-16*	(2429 vs. 3322) | (1434 vs. 551)	(24 vs 38) | (16 vs 5)
	Smoking History	No Smoking History | With Smoking History	P = 2e-16*	(3376 vs. 2434) | (512 vs. 1581)	(37 vs 32) | (5 vs 12)
TB Presentation	GeneXpert	Low Bacterial Load | High Bacterial Load	P = 2e-16*	(3998 vs. 858) | (3998 vs. 3198)	(44 vs 16) | (44 vs 29)
	Sputum Smear	Low Bacterial Load | High Bacterial Load	P = 0.2	(3998 vs. 631) | (3998 vs. 3173)	(44 vs 13) | (44 vs 28)
	Lung Cavity	No Cavity | With Cavity	P = 2e-16*	(3998 vs. 905) | (3998 vs. 3151)	(44 vs 18) | (44 vs 27)

Table S16. Statistical significance in comparing smartphone model performance for various sub-categories: P<0.05 marked with * indicate significant difference between the models using Delong test.
